# Increasing the precision of simulated percutaneous dilatational tracheostomy—a pilot prototype device development study

**DOI:** 10.1016/j.isci.2024.109098

**Published:** 2024-02-02

**Authors:** Athia Haron, Lutong Li, Eryl A. Davies, Peter D.G. Alexander, Brendan A. McGrath, Glen Cooper, Andrew Weightman

**Affiliations:** 1 School of Engineering, Faculty of Science and Engineering, The University of Manchester, Manchester, UK; 2 Greenlane Department of Cardiothoracic and ORL Anaesthesia, Auckland City Hospital, Auckland, New Zealand; 3 Manchester University NHS Foundation Trust, School of Biological Sciences, Faculty of Biology Medicine and Health, University of Manchester, Manchester, UK; 4 Manchester University NHS Foundation Trust, Manchester Academic Critical Care, Division of Infection, Immunity and Respiratory Medicine, School of Biological Sciences, Faculty of Biology Medicine and Health, University of Manchester, Manchester Academic Health Sciences Centre, Manchester, UK

**Keywords:** Health sciences, Surgery, Respiratory medicine

## Abstract

Percutaneous dilatational tracheostomy (PDT) is a bedside medical procedure which sites a new tracheostomy tube in the front of the neck. The critical first step is accurate placement of a needle through the neck tissues into the trachea. Misplacement occurs in around 5% of insertions, causing morbidity, mortality, and delays to recovery. We aimed to develop and evaluate a prototype medical device to improve precision of initial PDT-needle insertion. The Guidance for Tracheostomy (GiFT) system communicates the relative locations of intra-tracheal target sensor and PDT-needle sensor to the operator. In simulated “difficult neck” models, GiFT significantly improved accuracy (mean difference 10.0 mm, ANOVA p < 0.001) with ten untrained laboratory-based participants and ten experienced medical participants. GiFT resulted in slower time-to-target (mean difference 56.1 s, p < 0.001) than unguided attempts, considered clinically insignificant. Our proof-of-concept study highlights GiFT’s potential to significantly improve PDT accuracy, reduce procedural complications and offer bedside PDT to more patients.

## Highlights

• Our prototype device (GiFT) improved accuracy in simulated bedside tracheostomy (PDT)
• Performance was improved for novice and experienced operators
• GiFT increased procedural time, considered clinically insignificant
• GiFT may enhance safety, enabling more bedside tracheostomies, reducing delays

## Graphical abstract

**Figure:**
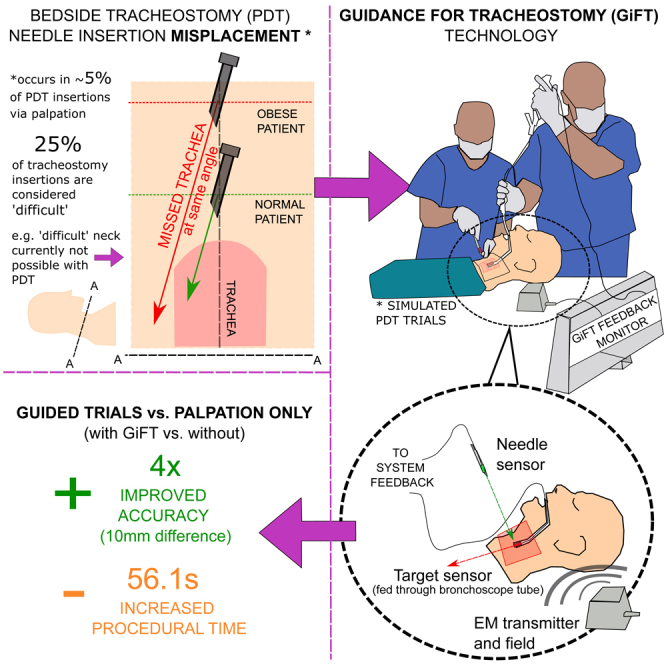

## Introduction

Tracheostomy involves creating a temporary or permanent communication between the front of the neck and the trachea and is often performed on the sickest and most complex patients. While timely insertion and optimal management has been shown to improve the safety and quality of care, significant risks are associated with new tracheostomy insertion.^1^^,^^2^^,^^3^ The classical “surgical” insertion procedure has been described for centuries, and modern approaches can negotiate complex neck anatomy and pathology, correctly identify the trachea and optimal level for tracheotomy, and deal with bleeding from large vessels (via ligature) or smaller vessels (via electrocautery). Bleeding is anticipated or realized in 10–12% of all procedures.^4^ Surgical tracheostomy almost always necessitates transfer to an operating theater with an appropriately trained surgeon, assistant, theater team and anesthetic team. One of the biggest challenges for surgical tracheostomy in the critically ill is coordinating a window of patient, surgeon and theater team availability. Delays and rescheduling of surgery risk complications, particularly if therapies such as anticoagulation, enteral nutrition and insulin need to be continually suspended and restarted.

The dominant historical indication for tracheostomy has been to bypass an actual or anticipated obstruction of the upper airway, typically caused by cancer, trauma or swelling. However, around two-thirds of procedures are performed to facilitate long-term ventilation and “weaning” from invasive respiratory support provided to critically ill patients.^1^^,^^5^ Tracheostomy allows sedation to be reduced or stopped, facilitates titration of ventilatory support, and can promote laryngeal recovery and rehabilitation.^6^ One of the drivers of this revolution in the utility of tracheostomy was the development of a safe, bedside procedure.^5^ Percutaneous dilatational tracheostomy (PDT) involves the use of the Seldinger technique: insertion of a needle through the neck into the trachea; passing a guidewire through the needle; passing dilators over the wire until the stoma is large enough to fit a suitable tube.^5^ PDT involves less dissection and cutting than surgical techniques, which in turn may cause less tissue trauma and bleeding. Significantly, PDT can be performed by appropriately trained intensive care specialist teams at the intensive care unit (ICU) bedside, removing the need to transport a critically ill patient to an operating theater and eliminating associated scheduling complexities. Timely tracheostomy can significantly reduce overall time spent requiring invasive ventilation which in turn is associated with a reduction in ventilator-associated pneumonia (VAP), delirium, and ICU and hospital length of stay.^7^

Around 25% of tracheostomy insertions are considered “difficult.”^8^ Most centers currently opt for a surgical tracheostomy if a patient has anticipated bleeding or if the trachea is not easily palpable; seen in around 30% of tracheostomy candidates with obesity or edema.^8^ Prior to the 2020 SARS-CoV-2 virus pandemic, tracheostomy could be anticipated in 8–13% of patients receiving advanced respiratory support in modern ICUs.^1^^,^^9^ Reported rates of tracheostomies utilized during the pandemic increased, but varied from 16 to 61%.^10^^,^^11^^,^^12^

PDT insertion can be complicated: there are important neck structures to avoid such as the head and neck blood vessels (carotid, innominate and subclavian arteries, jugular and anterior thyroid veins), nerves (vagus, brachial plexus, and recurrent laryngeal in particular), viscera and tissue (lungs and pleura, esophagus, thyroid, and thymus) and structures such as the thoracic duct; the trachea can be difficult to locate and puncture; the tracheal target typically has a 10 mm internal diameter, is mobile within the tissue planes of the neck moves with respiration and pulsation of adjacent major blood vessels; and the critically ill ventilator-dependent patient cannot tolerate long or repeated procedures.^5^ Tracheostomy complications account for half of all airway-related deaths and cases of brain injury in the ICU.^13^ Tracheostomy insertion is the highest risk elective procedure undertaken in the critically ill and insertion is associated with high levels of anxiety among staff.^5^

**Figure 1:**
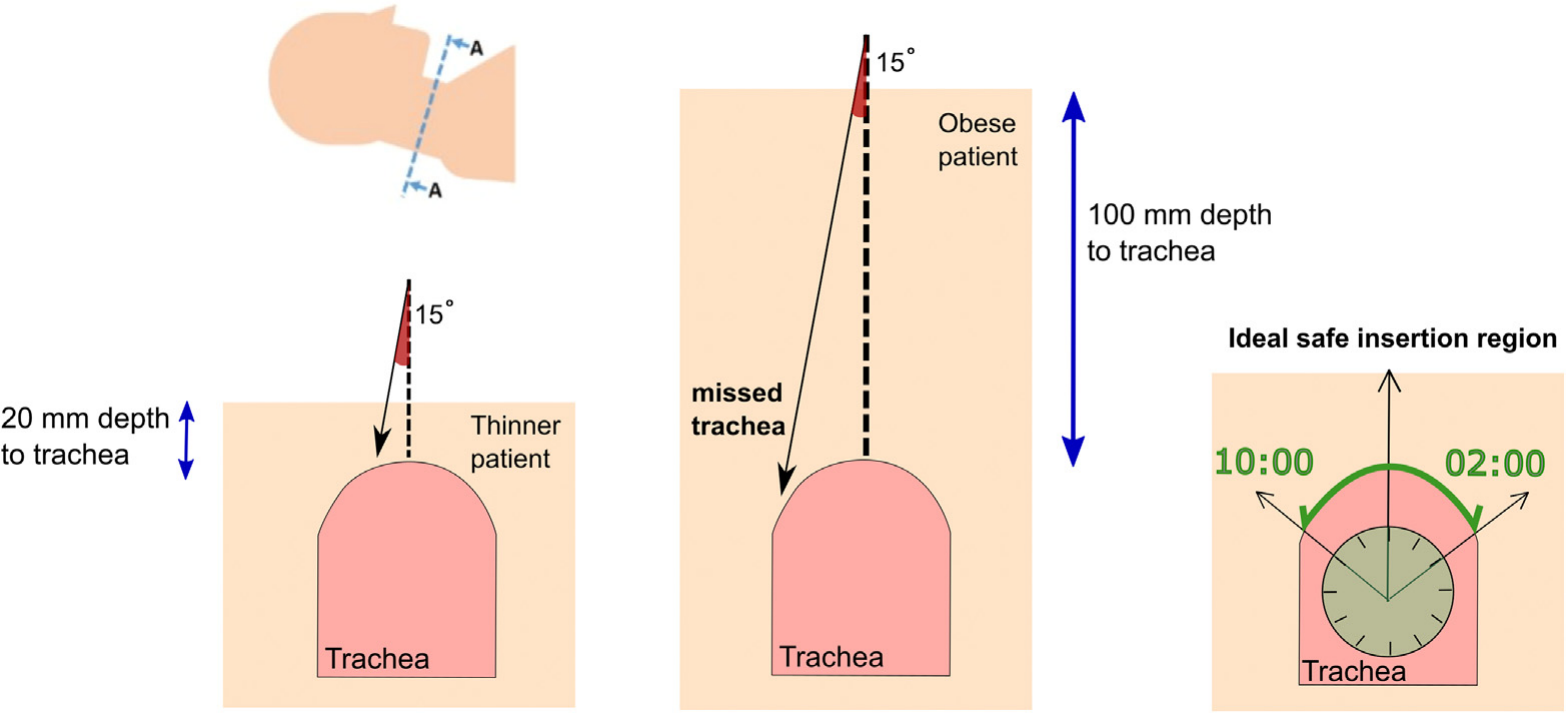
Schematic cross-sectional representation of the neck and trachea (bottom left image) demonstrating problems when attempting percutaneous tracheostomy in an obese patient (right image) compared with a thinner patient (bottom left image) Both PDT insertion attempts are inaccurate by 15° but the target trachea is still located by the black solid arrow (needle) in the left-hand image (although not at an optimal puncture site). The needle misses the trachea in the obese patient and risks damaging important adjacent structures in the neck. Image on the far right represents the ideal insertion angles of the PDT procedure. A clockface shows the “safe insertion angle” region between 10:00 to 02:00.

Current insertion techniques rely on palpation, clinical judgment and experience. Ultrasound of the neck can identify vessels prior to insertion, but real-time guidance is limited by the footprint and fidelity of the probes and the poor acoustic properties of the air-filled tracheal target. Endoscopic guidance is currently recommended, visualizing the trachea from the inside as the needle is advanced.^14^ However, endoscopy can only visualize the needle once it enters the trachea and offers no guidance beyond puncture confirmation. Virtual or augmented reality shows promise for the future, but no systems are currently available to help guide tracheostomy insertion beyond superimposing the bronchoscopic view into the operators’ field of vision or physically stabilizing the needle.^15^^,^^16^ Optimal initial tracheal needle puncture is in the midline between the second and third tracheal rings. Needle insertion based on palpation and dead reckoning alone risks missing not only the optimal insertion point, but missing the trachea altogether, especially as the distance from the skin to target increases (Figure 1). Transillumination techniques using the bronchoscope light to localize the puncture point have been used by some researchers and clinicians to provide guidance. This technique is has demonstrated limited success as a thin patient^17^ is needed, and a dark room (not ideal for a high-risk technically challenging, unguided procedure).

There remains an unmet need to improve the precision and safety of PDT. We describe the development and initial testing of a novel prototype medical device (Guidance for Tracheostomy; GiFT) with the potential to improve this common procedure. The obvious safety benefits of a precise, first pass, guaranteed puncture of the trachea at the optimum target site, even for patients considered technically difficult, are augmented by the potential to perform PDT at the ICU bedside for a proportion of those patients who would otherwise risk potential delays awaiting a surgical procedure. Especially for the obese, post-procedural complication occur 2–4 times more frequently with surgical tracheostomy.^18^

Our aim was to conceive, design, and evaluate in the simulated clinical environment a prototype medical device capable of precisely guiding an operator to accurately insert a needle into the trachea as a prelude to PDT. Our objectives were to demonstrate that the technology was usable, accurate, and feasible in the clinical environment, and to explore optimal user feedback for the guidance through evaluation of performance and participant questionnaires. We hypothesized that PDT undertaken with GiFT would have greater precision and would eliminate failure to enter the trachea during simulated PDT in an obese neck model.

## Results

Twenty consenting participants were recruited; ten laboratory and hospital staff. Clinical participants described a range of experience levels with seven consultants, two senior trainees, and one staff grade doctor. Six clinicians had been PDT lead operator more than 20 times.

Two attempts were not completed by different laboratory-based participants (one Haptic-Visual and Audio-Visual feedback). Hospital staff were a mean difference of 19.2 s faster and 0.3 mm more accurate across all feedback modalities than laboratory staff (Table 1). These differences were not significant.

**Table 1:** Differences between laboratory and hospital staff across all feedback modalities

	Staff group	Total procedural count	Mean	(SD)	Median	(IQR)	Mean difference	Wilcoxon p
Time to target (s)	Laboratory	78	97.2	(87.2)	66.2	(86.8)	19.2	0.232
	Hospital	80	78.0	(63.8)	56.3	(65.9)		
Radial distance to target (mm)	Laboratory	78	6.18	(7.38)	3.72	(4.30)	0.30	0.828
	Hospital	80	5.88	(7.30)	3.39	(3.24)		

**Figure 2:**
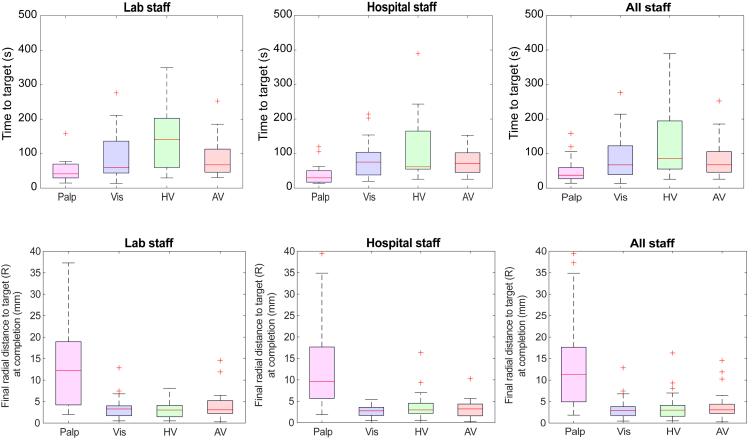
Boxplot Panel Time to target (top panel) and final radial distance to target at completion (bottom panel) separated into staff groups. Boxplots show raw data points, range, IQR and median values in each category. Abbreviations are used to indicate the feedback modalities: Palp, no GiFT feedback (palpation only); Vis, visual GiFT feedback only; HV, GiFT haptic and visual feedback; AV, GiFT audio and visual feedback. See Tables for significance testing.

There were significant differences between attempts made with GiFT versus without: radial distance to target (R) was a mean difference of 10.0 mm closer (p < 0.001) while time to target was a mean difference of 56.1 s slower (p < 0.001) when using any of the GiFT feedback modalities (Table 2 and Figure 2). A summary of observations of the different feedback modalities for all the participants can be found in Table 3.

**Table 2:** Differences in performance between all GiFT feedback modalities and no GiFT (palpation only)

		Total procedural count	Mean	(SD)	Median	(IQR)	Mean difference	ANOVA p (type III)
Time to target (s)	No GiFT	38	45.9	(30.6)	37.3	(32.1)	56.1	<0.001
	GiFT	120	102.0	(82.3)	74.5	(91.8)		
Radial distance to target (mm)	No GiFT	38	13.50	(10.8)	11.3	(12.7)	10.02	<0.001
	GiFT	120	3.48	(2.69)	2.98	(2.4)

**Table 3:** Summary of observations for the different feedback modalities for all participants

	Feedback modality	Count	Mean	(SD)	Median	(IQR)
Time to target (s)	Palpation	40	45.9	(30.6)	37.3	(32.1)
	Visual	40	88.6	(66.0)	67.3	(83.0)
	Haptic-visual	38	134.0	(111.0)	85.8	(139.4)
	Audio-visual	38	82.2	(49.0)	67.5	(59.7)
Radial distance to target at completion (mm)	Palpation	40	13.5	(10.8)	11.3	(12.7)
	Visual	40	3.18	(2.23)	2.87	(2.13)
	Haptic-visual	38	3.50	(2.87)	3.02	(2.55)
	Audio-visual	38	3.77	(2.96)	3.07	(2.17)

There were significant differences between the feedback modality groups for time to target (ANOVA F = 10.45, p < 0.001) and for radial distance to target (F = 28.97, p < 0.001). Multiple pairwise comparison showed that final radial distance to target was significantly smaller (more accurate) with all GiFT modalities versus no GiFT (all p < 0.001). Time to target was significantly slower with visual (p = 0.037) and haptic-visual (p < 0.001) feedback when compared to no GiFT. Group difference between each GiFT modality versus no GiFT can be found in Table S1 in the supplementary materials. The addition of haptic feedback generally increased time to target (Figure 2 *boxplot panel*).

### Questionnaire and feedback from participants

The questionnaire feedback supported the performance evaluation of the participants, and they were unaware of their performance metrics with respect to radial distance to target and time to target. The key findings from the questionnaire with clinical participants are summarized as follows:

Eight out of ten clinicians agreed that lack of physical cues/landmarks for difficult neck anatomy can lead to errors in PDTs. Clinicians felt that the GiFT system can improve confidence in performing PDT, for example *“… I like the reassurance it gives.*” While eight out of ten of participants were “almost always confident” that PDT procedures in real patients would be successful without any complications, the majority (6 out of 10 participants) was at least sometimes stressed when performing a PDT in the ICU. Visual only and visual-audio feedbacks were tied as most informative and most intuitive to use, while visual-haptic feedback was unanimously found to be least informative. The procedure with visual feedback of the GiFT system was found to be the easiest to perform compared to all other modalities (including no-GiFT). Two out of ten clinicians found the haptic feedback distracting and worried about the impact in precision during the procedure. The full results summary of the questionnaire can be found in supplementary materials as “*GiFT Clinicians Study Questionnaire*.”

## Discussion

Our study has demonstrated that guided needle insertion during simulation of an anatomically representative PDT procedure can significantly improve accuracy when compared to a palpation-only unguided technique. There were insignificant differences between laboratory and hospital staff on all feedback modalities and were not considered clinically relevant in the setting of PDT. Improvements in accuracy were considered clinically meaningful (a 10 mm mean difference when targeting a 10 mm diameter trachea) and came at the expense of a mean increase in overall procedural duration of 56 s (around one-quarter of the SD of time to target). Arguably, this is a clinically acceptable increase in procedural time associated with better accuracy and the potential to reduce procedural complications. The only clinically recorded standard deviation of procedural times for a PDT is around 67.1 s^19^ thus we consider the increase of procedural time using GiFT to be clinically insignificant and does not detract from the main goal of our study which was focused on determining the accuracy and success of using the system. Not to mention that, innovations in procedural guidance offer additional benefits to patients and to healthcare systems.

From the questionnaire, the system offered an intuitive method to perform the procedure, as the majority found the visual only feedback of the GiFT system to be the easiest to perform compared to all other modalities (including no-GiFT). There was no significant difference between any of the GiFT feedback modalities compared to no-GiFT (or palpation only). However, preference was noted and the haptic feedback in particular caused distraction in two clinicians. As we aimed to explore different feedback modalities, this was noted and is useful for future versions of the system in determining the best system feedback.

The GiFT system is focused on initial needle placement, which we consider the most critical and highest risk element of the PDT procedure. Using a precision target for needle insertion will influence other areas of the procedure, however. Operators are required to carefully withdraw the tracheal tube to facilitate bronchoscopy and placement of the tracheal target sensor, and as a result, we anticipate that the frequency of cuff puncture will reduce, as the needle is guided away from the cuff. Our system has no influence on later aspects of the procedure.

**Figure 3:**
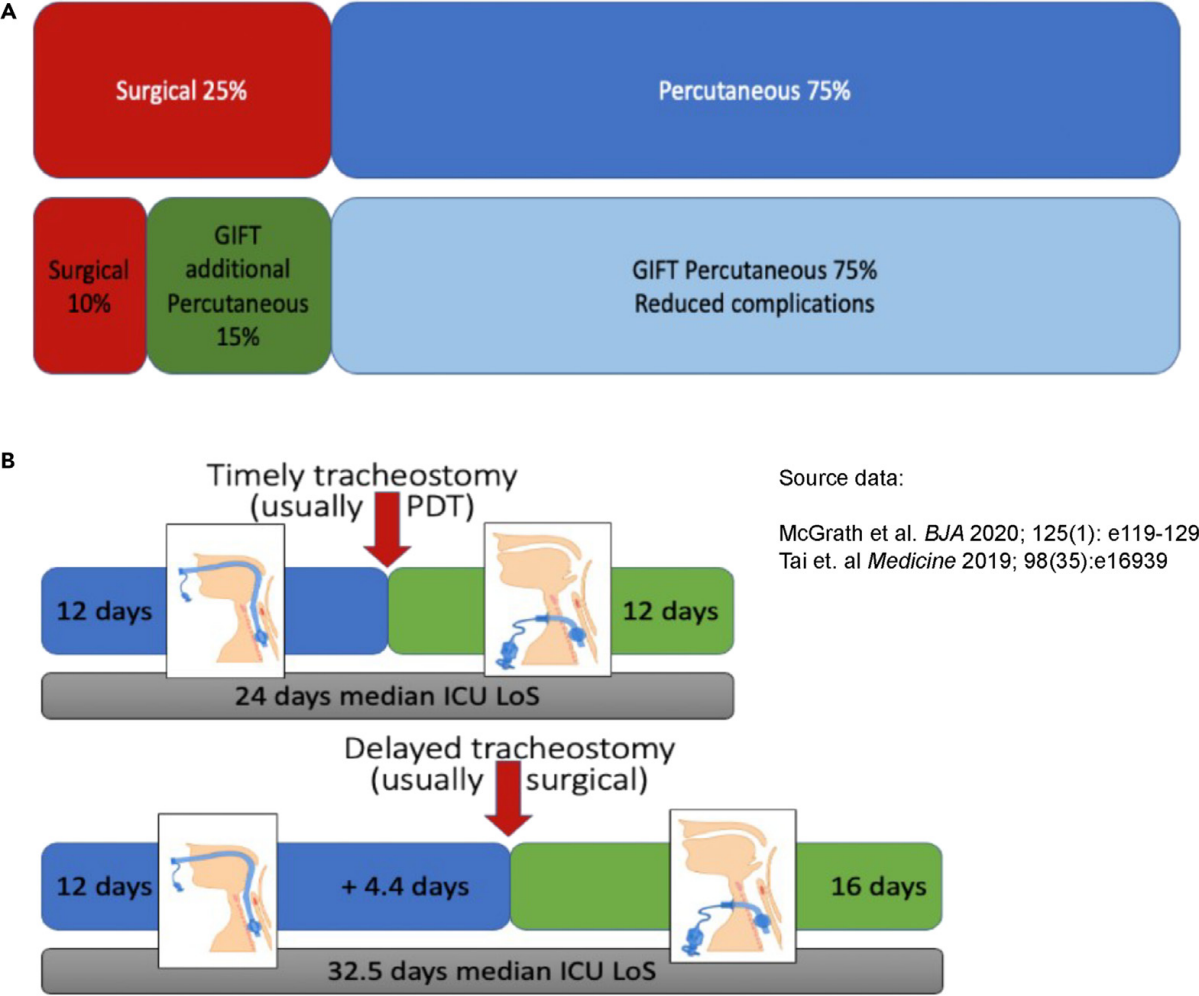
Illustration of how the implementation of GiFT system could reduce numbers of patients requiring a planned surgical procedure and the impact of delays of timely tracheostomy in the critically ill (A) Top row: current situation with 25% of cases considered difficult and/or with a bleeding risk. Bottom row: We hypothesize, based on our bench tests of the GiFT system that we report, that the implementation of GIFT could significantly reduce the numbers of patients requiring a planned surgical procedure (in addition to reducing complications). (B) The impact of delays to timely tracheostomy in the critically ill. Source data from Higgins and Punthakee.^23^.^2^ LoS, length of stay.

Tube misplacement is one of the most feared complications during tracheostomy insertion, resulting in loss of the airway and/or injury to surrounding structures.^20^ Misplacement is more common in the obese and when using a percutaneous technique.^21^^,^^22^ The GiFT system may reduce misplacement and associated complications, but also mean more challenging cases could be undertaken percutaneously at the bedside in the ICU (Figure 3A). A comparison of surgical versus percutaneous tracheostomy found PDT was less resource-intensive and less costly than surgical techniques (by an average of $456.61 USD).^23^ Tai’s 2019 study from Taiwan in 134 patients at a single center evaluated the tracheostomy delay time, defined as the time from the physician’s decision to undertake tracheostomy to the procedure.^24^ Overall, there was a mean delay of 4.4 days, heavily weighted by surgical tracheostomy, representing around 25% of mechanical ventilation time before tracheostomy. Delays were two and a half times more common when a surgical procedure was planned when compared with PDT (OR 2.49, 95%CI 1.1–5.9). Longer delays were significantly associated with a decreased weaning rate from mechanical ventilation, longer ICU and hospital stays, and increased mortality and reducing availability of ICU beds for other patients who might benefit.^25^ Prolonged oral intubation prior to tracheostomy increases consumable and drug costs and increases the risk of developing VAP.^6^^,^^26^^,^^27^^,^^28^ Attributable VAP mortality is estimated at 4.0–13.5%, with additional financial costa of a VAP episode estimated at £7,000–£25,000.^7^^,^^29^^,^^30^^,^^31^ There are significant burdens for the patient and their family of delayed tracheostomy, delaying waking, communication and recovery of laryngeal and other functional rehabilitation.^32^ Figure 3B depicts a graphical representation of the impact of delayed tracheostomy using data from Tai et al.^24^ and a recent UK tracheostomy Quality Improvement study.^2^

One of the challenges in translating a proof-of-concept to a useful medical device is integrating it into current workflows without increasing the burdens on staff or the system. Our participants favored simple visual-only feedback, even in the controlled environment of the experimental setting. Clinical PDT at the bedside of a complex, ventilator-dependent patient within an ICU presents additional constant audio and visual feedback from vital signs monitors, the ventilator, bronchoscopy screens, the practical procedures of airway management and the PDT itself. Integrating guidance into these existing systems and workflows may improve acceptance and usability, and novel methods of providing guidance and positional feedback for invasive procedures such as PDT should be further explored.

### Limitations of the study

Our study has several limitations. While we used an anatomically accurate manikin in a representative clinical environment, the model and the situation are very different from undertaking PDT in a clinical setting. However, our model successfully replicated a difficult PDT and experienced clinical staff did not puncture the trachea quickly or easily. While we did not change the skin of the mannequin between procedures, which could add positioning bias by participants looking at previous puncture holes, we reduced this bias by changing the position and orientation of the trachea underneath the skin before each procedure. Although our sample size was small, we have demonstrated that the guidance system is capable of increased accuracy at the expense of increased procedural time. There is likely a learning curve which may reduce procedural time, but the difference in performance between laboratory staff and experienced clinicians when using different feedback modalities was not significant. This suggests that the feedback is intuitive, although procedural familiarity was a (non-significant) advantage for hospital staff. Future work should address the feedback from clinical staff: exploring integrations with more familiar positioning information and including angular indications in the feedback system. The principles of our guidance system may be applied to other clinical procedures where a target sensor can be placed via a natural or artificial body orifice.

### Conclusions

Tracheostomy is a common and important procedure in modern critical care but is associated with procedural complications and significant delays if a bedside percutaneous technique cannot be safely undertaken. Our proof-of-concept study has demonstrated that a prototype guidance system can significantly improve accuracy of PDT needle puncture during controlled experimental insertion attempts at the cost of modest increases in procedural time. Our study has clinical relevance, and the prototype system has the potential to positively influence procedural complications and the patient journey in critical care when tracheostomy is required. Further research is necessary to develop guidance technologies and to integrate them into procedural workflows to maximize potential benefits. Additionally, the follow-on research should consider the validation of the prototype system in animal models (e.g., porcine) or human cadavers, followed by assessments in adverse events of the puncture such as tracheal tube cuff puncture, posterior tracheal wall puncture and bleeding.

## STAR★Methods

### Key resources table

**Table:** 

REAGENT or RESOURCE	SOURCE	IDENTIFIER
**Deposited data**		

Experimental data	This paper	N/A

**Software and algorithms**		

R Studio v1.4.1106 (RStudio, Boston, US).	RStudio, Boston, US	N/A
Qt software (in C++ language),	Qt Company, Espoo, Finland	N/A
trackSTAR^TM^ software	NDI Medical, Ontario, Canada	N/A
Qualtrics XM	Qualtrics International Inc., Provo, Utah and Seattle, Washington, USA	N/A

### Resource availability

#### Lead contact

Further information and requests for resources and reagents should be directed to and will be fulfilled by the lead contact, Athia Haron (athia.haron@manchester.ac.uk).

#### Materials availability

This study did not generate new unique reagents.

#### Data and code availability

• All data reported in this paper will be shared by the lead contact upon reasonable request.
• Original code cannot be deposited in a public repository as this is related to the patent we have (see declaration of competing interests).
• Any additional information required to reanalyse the data reported in this paper is available from the lead contact upon request

### Experimental model and study participant details

To test our hypothesis, we designed an experiment with two groups of participants: lab staff with no clinical experience in order to simulate a group with complete tracheostomy novice, and hospital staff with experience of at least ten PDT procedures as the primary operator. The involvement of lab staff in this study was for gathering useful insight into the technology’s usability and effectiveness. We aimed to design a system that was simple and intuitive, and did not need significant experience and understanding of the PDT procedure to perform.

In order to undertake PDT as the primary operator, hospital staff will have been trained and exposed to a number of PDT procedures as the non-primary operator, and be considered competent to undertake the procedure with at least remote supervision. The NHS Health Research Authority and the University of Manchester research decision tools confirmed that this study did not require formal Research Ethical Approval, but the study was conducted under the governance of the University of Manchester Academic Critical Care Research Group. Consenting participants were shown a standardized presentation describing the rationale and conduct of the experiment, and given a demonstration of the equipment and test model.

### Method details

#### The guidance for tracheostomy system

Our PDT guidance system (see below figure) uses technology embedded into Original Equipment Manufacturer instruments already used for the procedure. The system uses the 3D Guidance trackSTAR tracking technology (NDI Medical, Ohio, USA) providing real-time (255Hz) positions (X, Y, Z) and angles (roll, pitch and yaw) of up to four sensors relative to the low power electromagnetic field (EM) produced by the transmitter. Two sensors were used: one placed in the tip of the PDT-needle, and the other in the bronchoscope working channel as the *target sensor*. This gave the relative positions and orientations of the PDT-needle and the target in the EM field (see below figure). These known positions and angles were used to calculate the guidance parameters, namely (1) the radial distance to target (R), (2) The angle between the coronal plane and the *PDT-needle sensor* (α) and (3) the distance to target (DTT) between the *PDT-needle sensor* and the *bronchoscope target sensor*, using vector mathematics (see below figure).

**Figure:**
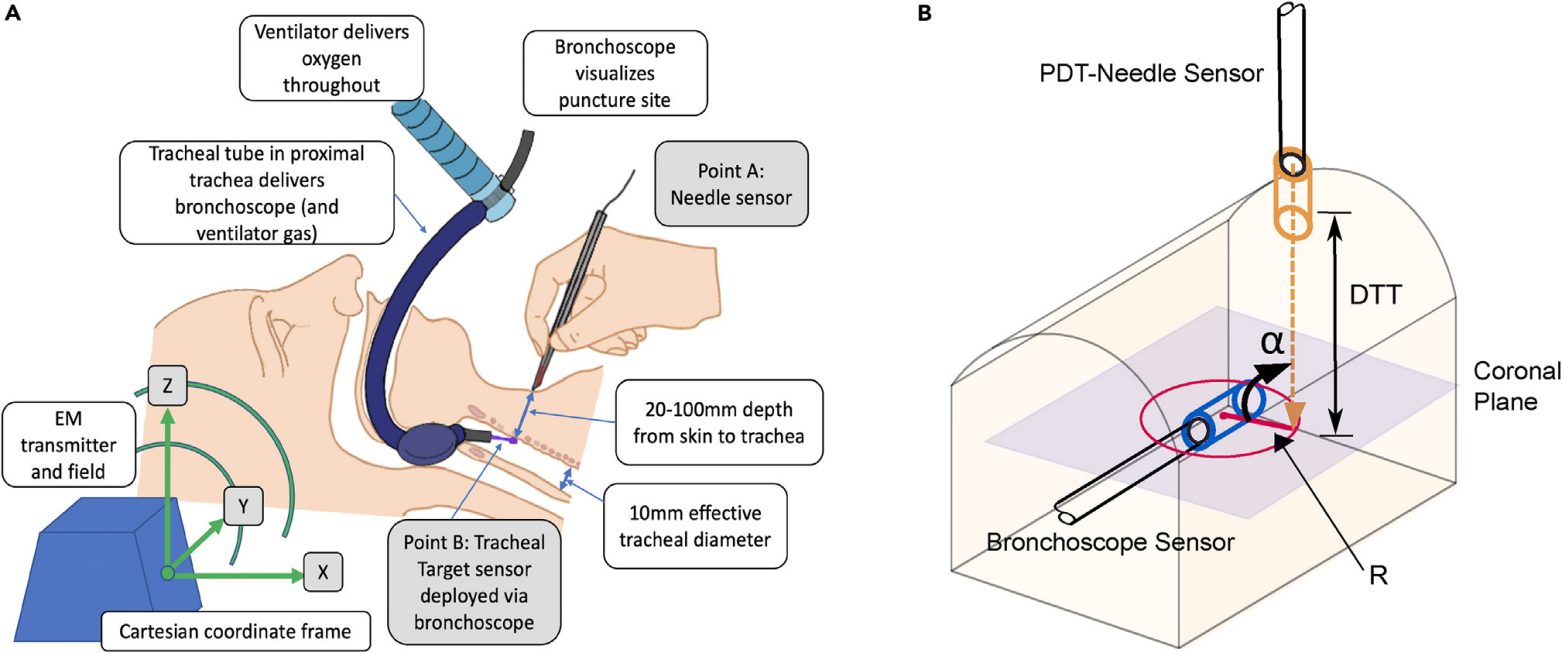
Ilustration of a percutaneous dilatational tracheostomy with GiFT hardware and the guidance parameters provided by the software (A) A schematic illustration of a percutaneous dilatational tracheostomy with GiFT hardware components; electromagnetic transmitter, *PDT-needle sensor*, *bronchoscope target sensor*. The cartesian coordinate frame (X, Y, Z) of the electromagnetic transmitter is illustrated, the system calculates the position (X, Y, Z) and orientation of coordinate frames attached to the *PDT-needle sensor* (point A) and the *bronchoscope target sensor* (point B). (B) Illustrating the guidance parameters (1) the radial distance to target (R), (2) The angle between the coronal plane and the *PDT-needle sensor* (α) and (3) the depth to target (DTT) calculated by the GiFT system and presented to the user.

The guidance parameters, calculated through software written in C++, were presented to the clinician undertaking the procedure via the GiFT UI, see below figure. The UI was developed through Qt software (in C++ language), incorporating the trackSTAR software and vector calculations. A user centered design approach was adopted to develop the UI, which has been shown to determine the success/failure of medical technologies.^33^ We finalized the UI via feedback and preliminary testing with clinical staff experienced in the PDT procedure. The GiFT UI presented guidance parameters to the clinician through a computer monitor collocated with the bronchoscope screen which is used as standard for the PDT procedure.

**Figure:**
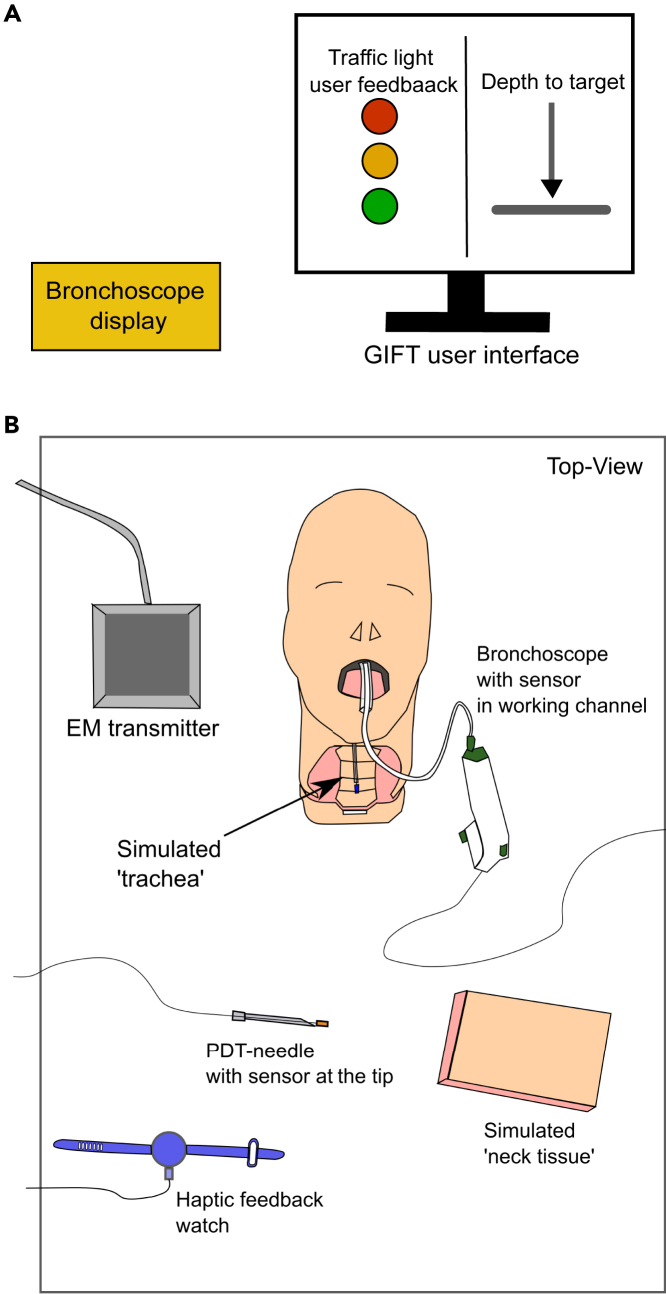
Illustration of the bronchoscope and GiFT feedback display used for the PDT procedures and the experimental setup of GiFT for the procedures (A) Illustrating the standard bronchoscope (aScope4, Ambu, Ballerup, Denmark) display utilized for the PDT procedure and the GiFT user interface. (B) Illustrating the experimental set up of the GiFT system. An anatomical model utilized for training was utilized with a simulated trachea manufactured from silicone skin simulant, additional silicone skin was placed over the Trachea. The trackSTAR EM transmitter, PDT-needle with sensor, Bronchoscope with sensor deployed through channel, haptic feedback watch, are illustrated.

The right-hand side of the UI presented a visual indicator illustrating the distance, in mm, of the *PDT-needle sensor* to the *bronchoscope target sensor*. The left-hand side of the UI presented information (see above figure) combining both the radial distance to target (R) and the angle between the coronal plane and the *PDT-needle sensor* (α). From the preliminary test with two experienced clinicians to determine the method for presenting guidance information, we found that presenting the radial distance to the target visually was more intuitive than other visual representations, such as using multiple axes.

Traffic light feedback was used to indicate if the position and orientation of the PDT-needle relative to the *bronchoscope target sensor* was suitable to hit the target.

The traffic light feedback presented to the clinician was described as: red as ‘do not continue with entry’; amber as ‘continue with entry, but with caution’; and green as ‘safe to continue with entry’. We developed the maximum safety margins of our system from the region of safe angle entry of 10:00 to 02:00 of a clock face in Figure 1. To aid the increase the accuracy of the procedure, we limited these margins even further. The final margins were as follows:

The margins for the red indicator was a radial distance to target >4.0 mm and the angle between the coronal plane and the *PDT-needle sensor* (α) > ± 10°, the amber indicator was a distance between 2.6 mm–4.0 mm, and α angle < ± 10°, and the green indicator was a distance of ≤2 mm and α angle of < ± 10°. This was to guide the operator to move the needle to reduce the radial distance to target using intuitive visual indicators. As such, going from red (not ready to proceed puncture) to green light (safe to proceed puncture) directly corresponds to a decrease in radial distance to the target.

In addition to the traffic light display of the visual feedback, the system provides feedback via audio and haptics. Using the same margins as the traffic light system, the audio system provides a beeping sound that increases in pitch by 25 Hz (from 470 Hz initial frequency) as the PDT-needle moves toward the correct position. While the haptic feedback decreases in vibration intensity until the PDT-needle is in the correct position, where the vibration stops completely (vibration intensity of ‘Buzz-2 80%’ waveform intensity to 0% waveform intensity, Adafruit DRV2605L Waveform documentation, Carter Nelson). The haptic feedback watch is worn at the non-procedural hand of the participant as to not affect the stability of the procedural hand that is navigating the PDT needle.

The method for presenting guidance information to users of the system was determined through preliminary testing with two clinicians with significant experience in PDT. Presenting the radial distance to target visually seemed more intuitive than other visual representations, such as multiple axes.

#### The experiments

The anatomical model (Airsim Advance Combo Bronchi, Adam Rouilly, Kent, UK) was orally intubated with a size 8.0mm *trans*-laryngeal tracheal tube (Shiley Evac tubes with TaperGuard cuff, used in routine clinical practice), terminating just below the model’s vocal cords. A bronchoscope was inserted via the tracheal tube and secured so that the target insertion point could be clearly visualized. The *bronchoscope target sensor* was advanced through the working channel of the bronchoscope to emerge into the bronchoscope’s field of view. The bronchoscope was not used to aid the navigation of the needle insertion itself, but was merely a tool for the placement of the target sensor in the trachea, and as a visualization tool to provide knowledge and confirmation of the insertion results. The exact location of the target was adjusted off-centre between attempts, out of the view of participants. The model simulated an impalpable trachea in an obese neck using simulated soft tissue to a depth of 25.0mm. In a typical thinner neck patient, the depth between the surface of the neck and the trachea is 20mm, in obese patients it can be as much as 100mm. In this study we choose a simulated depth of 25mm to provide favourable conditions for participants to estimate location without guidance, allowing comparisons drawn to be weighed against the GiFT technology.

Needle insertion was performed using a standard PDT kit (ULTRAperc PDT, Smiths Medical Ltd, Kent, UK) with the *PDT-needle sensor* passing internally via the hollow needle, securing the sensor at the needle tip. The second figure in the guidance for tracheostomy system illustrates the experimental set equipment utilized. Participants undertook a total of eight insertion attempts; two attempts with each of four feedback modalities.

1. No GiFT feedback (palpation only)
2. Visual GiFT feedback only
3. Audio and Visual GiFT feedback
4. Haptic and Visual GiFT feedback

The order of attempts was randomized using an online randomizer tool (http://www. randomizer.org). For each attempt, the trachea is randomly readjusted in position and angle to reduce effect of potential repetition bias. The primary outcome measures were *Time to Target* in seconds (measured by an observer from the first needle puncture of the skin to PDT needle visualized entering the trachea) and Radius from *Target* in millimeters (the distance from the *PDT-needle sensor* to the *bronchoscope target sensor* (tracheal) at the completion of the attempt. Distance was recorded by the GiFT device. Secondary outcomes included the number of attempts (defined as complete removal and re-insertion of the PDT needle from the model) and whether an attempt was abandoned (declared by the participant or by reaching a pre-determined 5-min time cut off). Following completion, hospital staff participants completed an online questionnaire (see supplementary material) exploring prior experience of PDT, complications, and their impression of the different feedback modalities.

#### The questionnaire

The online questionnaire was made using a university-preferred survey tool *Qualtrics XM* (Qualtrics International Inc., Provo, Utah and Seattle, Washington, USA). A total of 30 questions were asked. These were mixed-methods questions: i.e., multiple choice, Likert scales, rankings, and written feedback. We asked questions regarding their confidence in performing a successful PDT and possible causes of complications, particularly failed needle insertions. We also collected feedback comparing their experiences in performing the simulated PDT with and without the GiFT system; and what can be improved for all modalities. The results were summarized using the tool’s default report export (see supplementary material).

### Quantification and statistical analysis

Data were summarized into Microsoft Excel (Microsoft Corp., Washington, USA) tables and analyzed using R Studio v1.4.1106 (RStudio, Boston, US). Distributions were examined with appropriate plots and (Shapiro-Wilk) tests of normality. One or two-way ANOVA tests were used to compare group differences with Tukey HSD multiple pairwise comparisons made between the groups. Benjamini-Hochberg procedure adjusted p values for multiple comparisons. Type III sum-of-squares ANOVA test was used if groups were unbalanced. Levene’s test of homogeneity of variance led to Welch one-way tests if appropriate. Finally, given the sample size was anticipated to be significantly greater than 30, violations of assumptions of normality were accepted. The non-parametric Kruskal-Wallace test was used to confirm significance levels were not affected by these assumptions. Results are reported as mean (standard deviation), with 95% confidence intervals where appropriate, accepting a two-tailed p value < 0.05 as significant.

## Supplemental information

Document S1. Table S1

GiFT Experiment intro
