# Supplementary material for: Increasing the precision of simulated percutaneous dilatational tracheostomy—a pilot prototype device development study
Source: iScience. 2024 Feb 2;27(3):109098. doi: 10.1016/j.isci.2024.109098 (PMC10877963; doi:10.1016/j.isci.2024.109098)
Supplement: Document S1. Table S1 [file mmc1.pdf]

iScience, Volume *27*

Supplemental information

Increasing the precision of simulated percutaneous dilatational tracheostomy—a pilot prototype device development study

Athia Haron, Lutong Li, Eryl A. Davies, Peter D.G. Alexander, Brendan A. McGrath, Glen Cooper, and Andrew Weightman

Table of Contents

Table S1. Group differences between the different GiFT feedback modalities described as time and distance to target. Significant differences are indicated with an asterisk. 1

GiFT Clinicians Study Questionnaire .... 2 - 33

Table S1. Group differences between the different GiFT feedback modalities described as time and distance to target. Significant differences are indicated with an asterisk.

	Difference between group means	95% CI			Adjusted Tukey HSD p value
**Time to target (secs)**					
Vis-Palp	42·77	1·81	to	83·74	0·037*
HV-Palp	88·46	47·24	to	129·69	<0·001*
AV-Palp	36·36	-4·86	to	77·59	0·105
HV-Vis	45.69	4·46	to	86.92	0·023*
AV-Vis	-6·41	-47·64	to	34·82	0·978
AV-HV	-52·10	-93·59	to	-10·61	0·007*
**Distance to target (mm)**					
Vis-Palp	-10·362	-13·792	to	-6·932	<0·001*
HV-Palp	-10·039	-13·491	to	-6·587	<0·001*
AV-Palp	-9·776	-13·228	to	-6·324	<0·001*
HV-Vis	0·323	-3·129	to	3·775	0·995
AV-Vis	0·586	-2·866	to	4·038	0·971
AV-HV	0·263	-3·211	to	3·736	0·997

GiFT Clinicians Study Questionnaire

Exported as Qualtrics default report. June 5th 2023, 8:58 am MDT

Q1 - How many PDT procedures have you been the lead operator on?

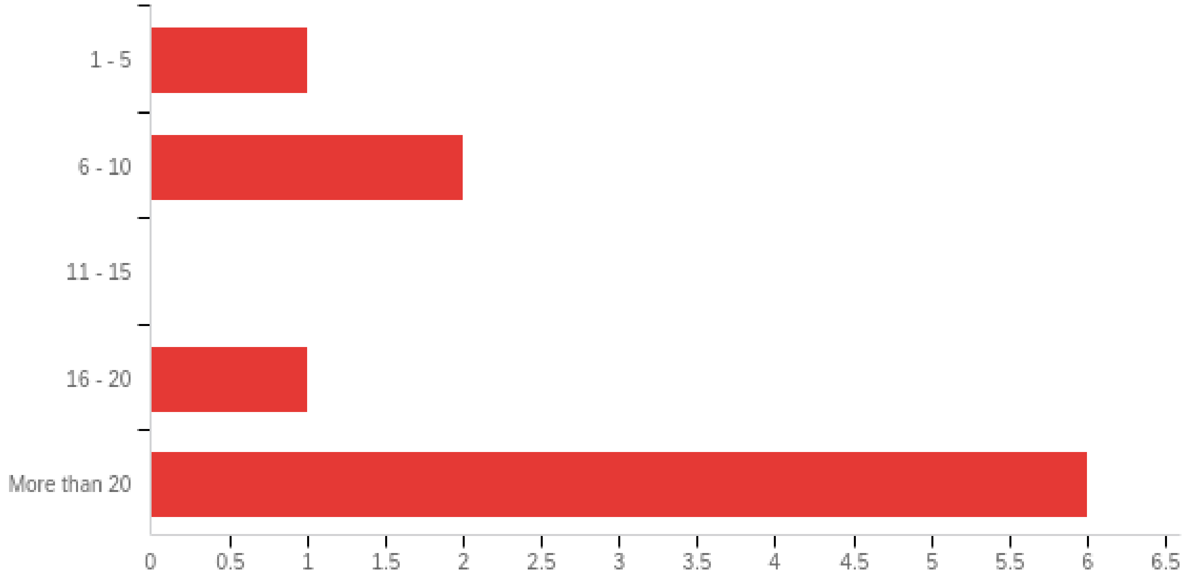

#	Field	Minimum	Maximum	Mean	Std Deviation	Variance	Count
1	How many PDT procedures have you been the lead operator on?	4.00	8.00	6.90	1.51	2.29	10

#	Answer	%	Count
4	1 - 5	10.00%	1
5	6 - 10	20.00%	2
6	11 - 15	0.00%	0
7	16 - 20	10.00%	1
8	More than 20	60.00%	6
	Total	100%	10

Q2 - How often are you confident that you will perform a successful PDT, where the ventilation tube has been inserted and the patient was stable after the procedure?

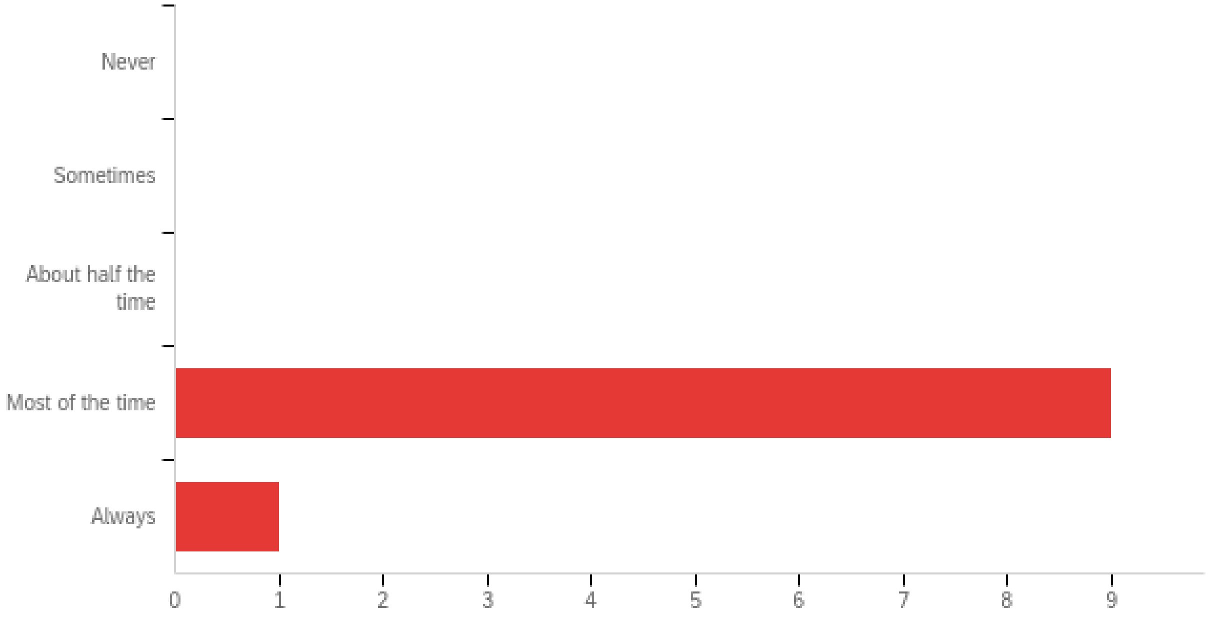

#	Field	Minimum	Maximum	Mean	Std Deviation	Variance	Count
1	How often are you confident that you will perform a successful PDT, where the ventilation tube has been inserted and the patient was stable after the procedure?	18.00	19.00	18.10	0.30	0.09	10

#	Answer	%	Count
15	Never	0.00%	0
16	Sometimes	0.00%	0
17	About half the time	0.00%	0
18	Most of the time	90.00%	9
19	Always	10.00%	1
	Total	100%	10

Q3 - How often are you confident that the procedure will be successful, without any complications from the procedure?

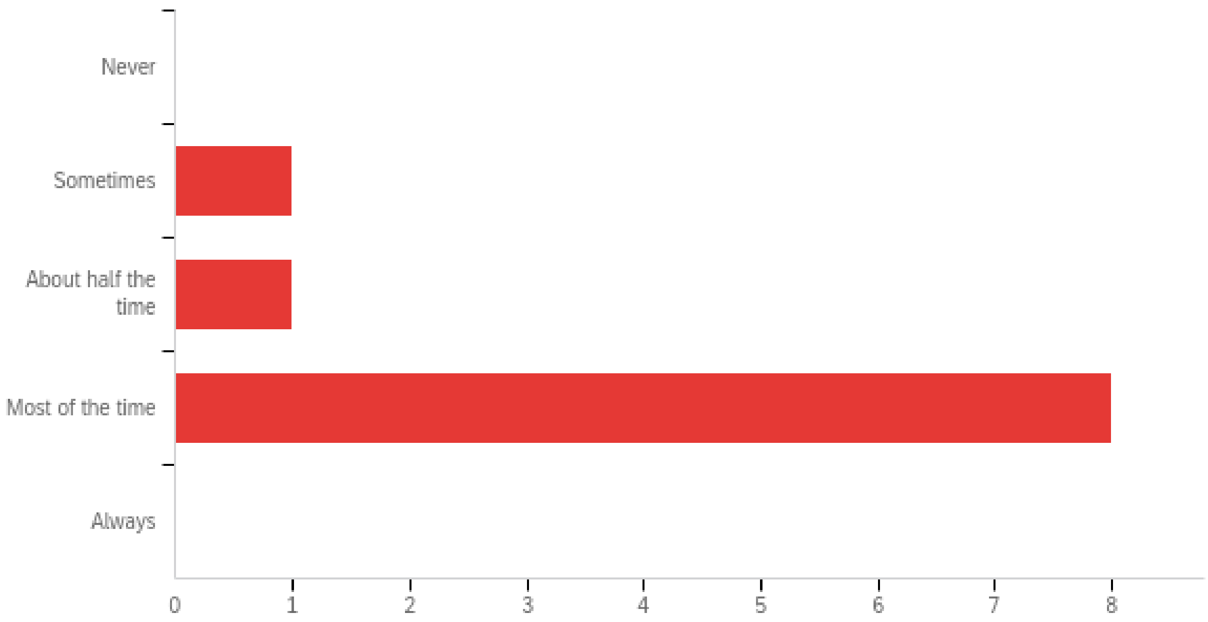

#	Field	Minimum	Maximum	Mean	Std Deviation	Variance	Count
1	How often are you confident that the procedure will be successful, without any complications from the procedure?	25.00	27.00	26.70	0.64	0.41	10

#	Answer	%	Count
24	Never	0.00%	0
25	Sometimes	10.00%	1
26	About half the time	10.00%	1
27	Most of the time	80.00%	8
28	Always	0.00%	0
	Total	100%	10

Q4 - How often are you stressed when performing a PDT in the ICU?

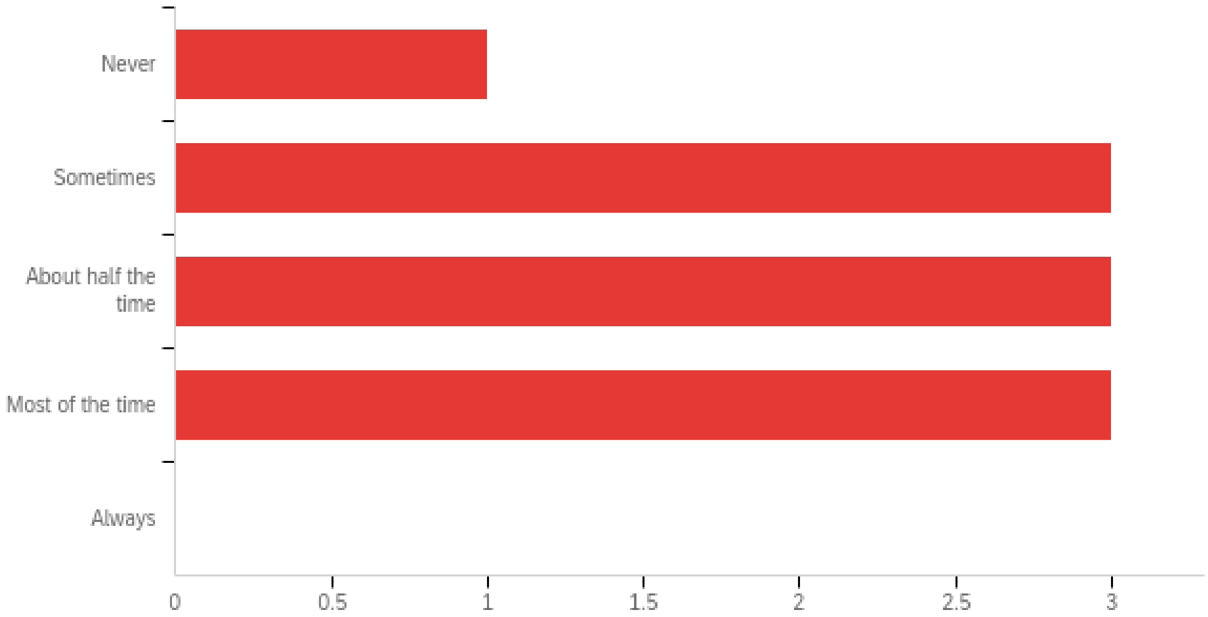

#	Field	Minimum	Maximum	Mean	Std Deviation	Variance	Count
1	How often are you stressed when performing a PDT in the ICU?	61.00	64.00	62.80	0.98	0.96	10

#	Answer	%	Count
61	Never	10.00%	1
62	Sometimes	30.00%	3
63	About half the time	30.00%	3
64	Most of the time	30.00%	3
65	Always	0.00%	0
	Total	100%	10

Q5 - What approximate percentage of successful initial PDT needle punctures would you say you have performed? (e.g., if you have done two and failed in one, your percentage is 50%). 'Success' here is defined as needle was inserted at the correct position, with no complications due to the insertion, and has not been reinserted more than 3 times).

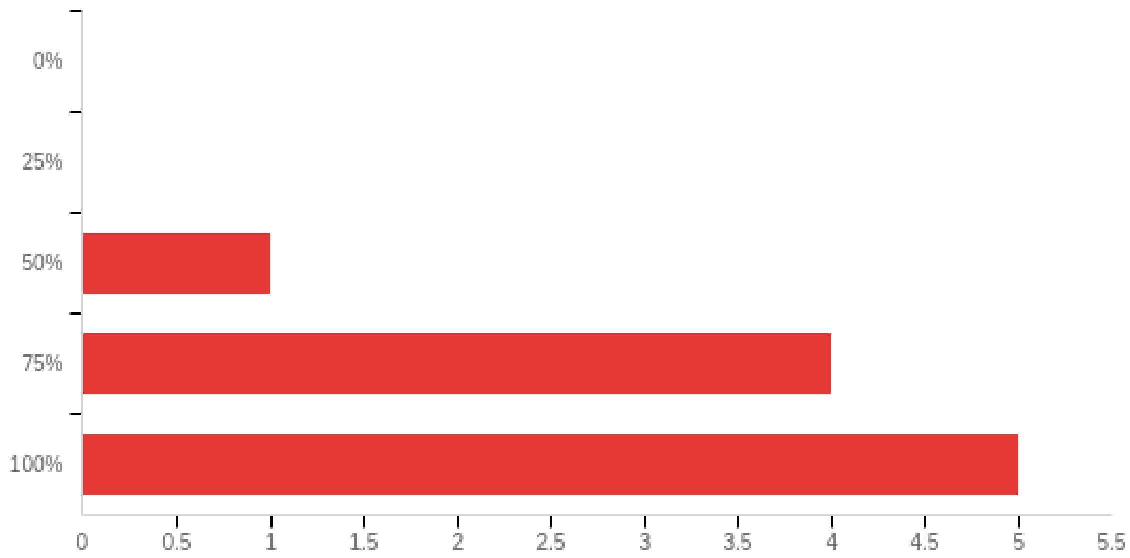

#	Field	Minimum	Maximum	Mean	Std Deviation	Variance	Count
1	What approximate percentage of successful initial PDT needle punctures would you say you have performed? (e.g., if you have done two and failed in one, your percentage is 50%). 'Success' here is defined as needle was inserted at the correct position, with no complications due to the insertion, and has not been reinserted more than 3 times).	3.00	5.00	4.40	0.66	0.44	10

#	Answer	%	Count
1	0%	0.00%	0
2	25%	0.00%	0
3	50%	10.00%	1
4	75%	40.00%	4
5	100%	50.00%	5
	Total	100%	10

Q6 - Have you performed a PDT that resulted in an immediate complication? If yes, how often does this occur, and what is the nature of this complication?

Have you performed a PDT that resulted in an immediate complication? If yes, how often does this occur, and what is the nature of this complication?
No
no
Bleedingm- rare
No
No
No
No
No
No
Yes - bleeding, manifested when local anaesthetic with adrenaline wore off afterwards

Q7 - How distracting is the surrounding environment in the ICU (e.g. machine sounds, other patients, etc.) where you are performing the PDT?

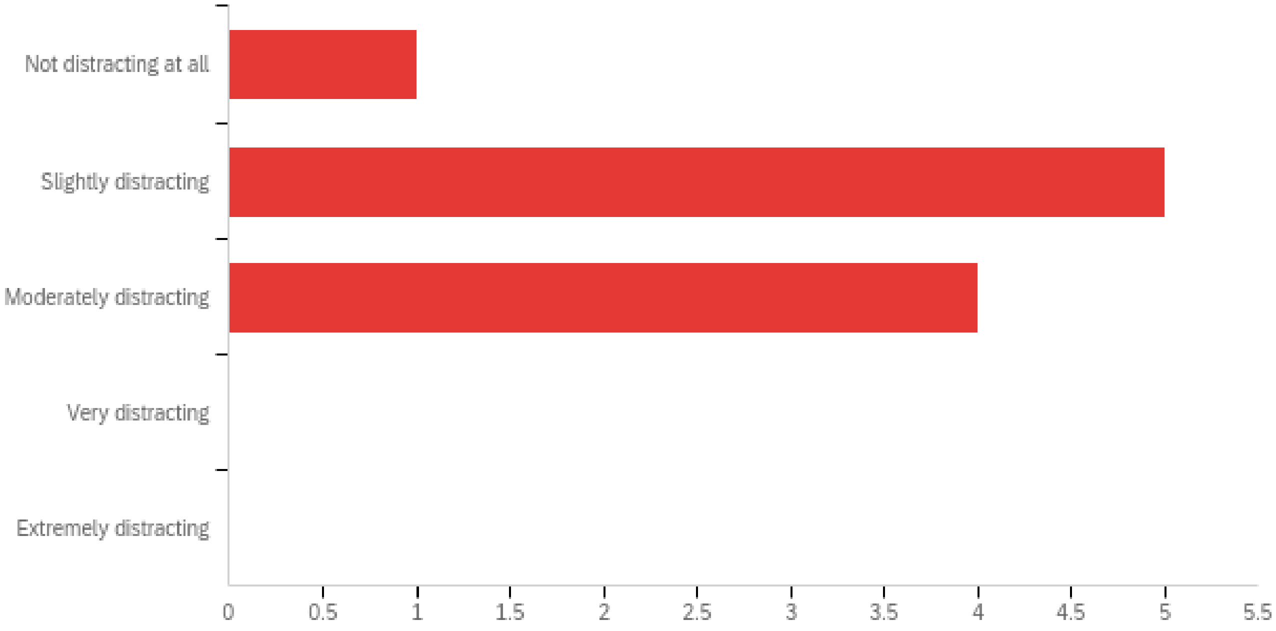

#	Field	Minimum	Maximum	Mean	Std Deviation	Variance	Count
1	How distracting is the surrounding environment in the ICU (e.g. machine sounds, other patients, etc.) where you are performing the PDT?	21.00	23.00	22.30	0.64	0.41	10

#	Answer	%	Count
21	Not distracting at all	10.00%	1
22	Slightly distracting	50.00%	5
23	Moderately distracting	40.00%	4
24	Very distracting	0.00%	0
25	Extremely distracting	0.00%	0
	Total	100%	10

Q8 - Can you describe the factors that can be distracting in the ICU?

Can you describe the factors that can be distracting in the ICU?
patient instability during procedure, equipment failure
interruptions for decisions/ plans - via telephone/ vocera/ bleep
noise
Being interrupted during procedure
Noise, other patients and staff
Bleeps, lack of equipment
General noise, concerns with other patients
Noise, other clinical events
People moving around, excessive equipment by the bedside, noises from other bed spaces, staff talking not about the procedure

Q9 - If you had complications during a PDT, how often were they due to failed needle insertion

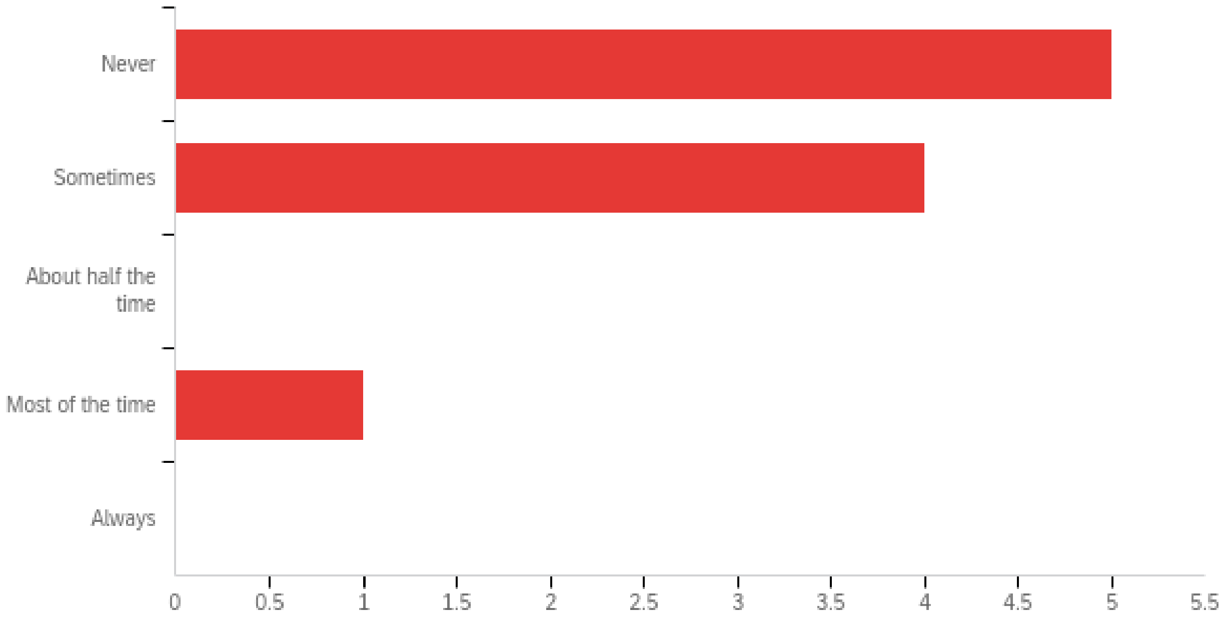

#	Field	Minimum	Maximum	Mean	Std Deviation	Variance	Count
1	If you had complications during a PDT, how often were they due to failed needle insertion	11.00	14.00	11.70	0.90	0.81	10

#	Answer	%	Count
11	Never	50.00%	5
12	Sometimes	40.00%	4
13	About half the time	0.00%	0
14	Most of the time	10.00%	1
15	Always	0.00%	0
	Total	100%	10

Q10 - During the needle insertion part of the procedure, have you ever inserted the needle at the right position only ONCE? If not, how many times do you usually need to reinsert the needle?

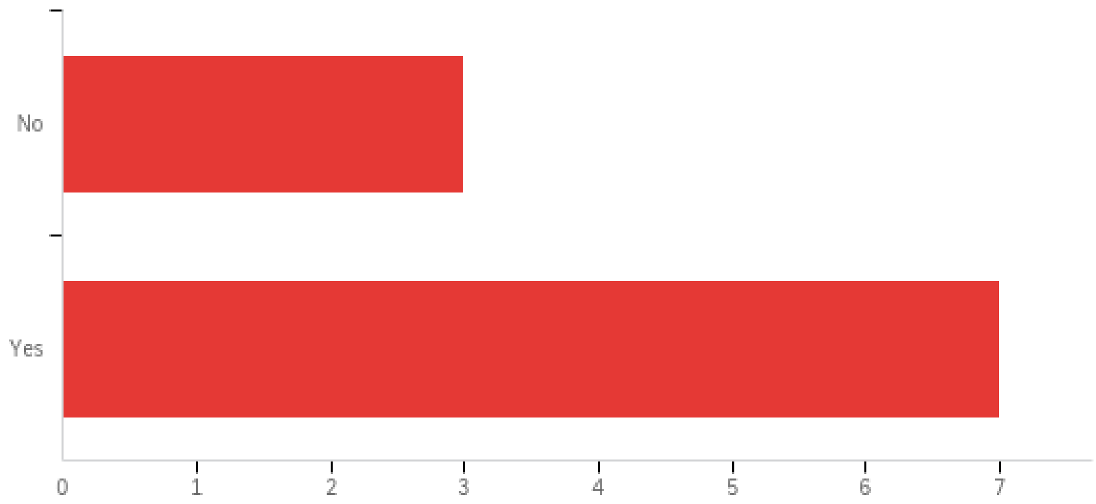

#	Field	Minimum	Maximum	Mean	Std Deviation	Variance	Count
1	During the needle insertion part of the procedure, have you ever inserted the needle at the right position only ONCE? If not, how many times do you usually need to reinsert the needle? - Selected Choice	21.00	22.00	21.70	0.46	0.21	10

#	Answer	%	Count
21	No	30.00%	3
22	Yes	70.00%	7
	Total	100%	10

Q10_21_TEXT - No

No - Text
1 - 2
2
3

Q11 - How likely, if you had a failed needle insertion, that it was due to lack of visual/physical cues for the insertion point?

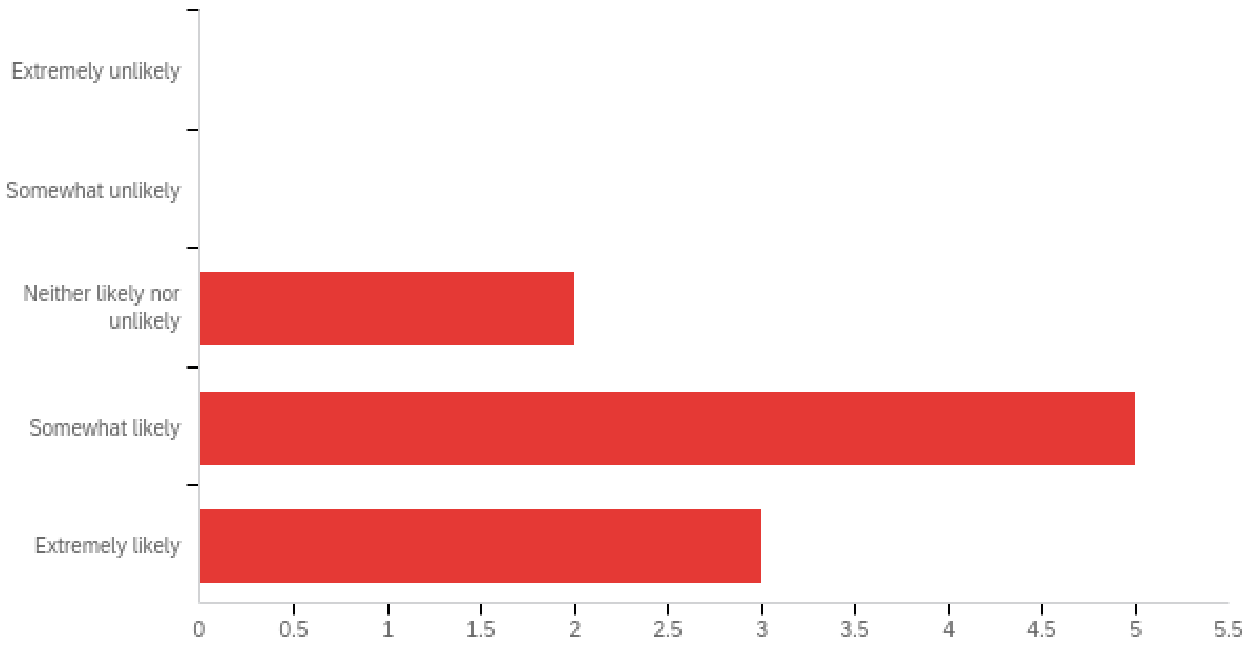

#	Field	Minimum	Maximum	Mean	Std Deviation	Variance	Count
1	How likely, if you had a failed needle insertion, that it was due to lack of visual/physical cues for the insertion point?	13.00	15.00	14.10	0.70	0.49	10

#	Answer	%	Count
11	Extremely unlikely	0.00%	0
12	Somewhat unlikely	0.00%	0
13	Neither likely nor unlikely	20.00%	2
14	Somewhat likely	50.00%	5
15	Extremely likely	30.00%	3
	Total	100%	10

Q12 - Could you describe other factors that can lead to a failed needle insertion?

Could you describe other factors that can lead to a failed needle insertion?
poor visualisation with bronchoscope, bleeding into trachea, poor positioning of patient
optimal postioning
overweight necks
Big neck, unable to palpate rings
Big neck
ET tube not withdrawn enough, lack of bronchoscope view of the needle insertion
Patient anatomy
Difficult anatomy

Q13 - From the introductory video, did you understand the aim of the study?

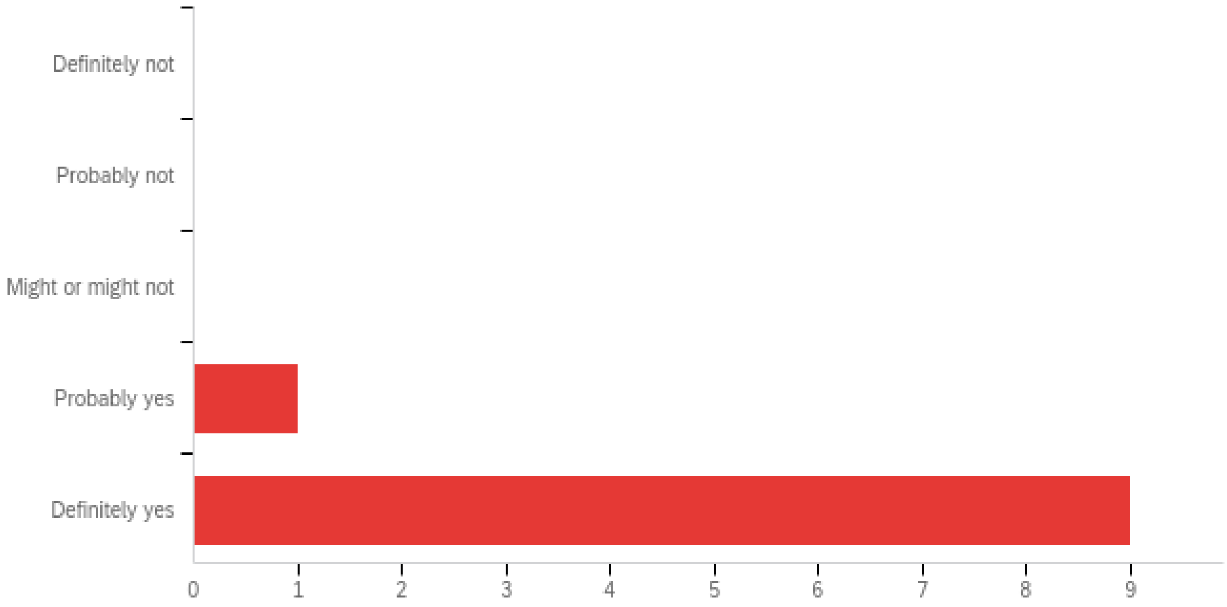

#	Field	Minimum	Maximum	Mean	Std Deviation	Variance	Count
1	From the introductory video, did you understand the aim of the study?	4.00	5.00	4.90	0.30	0.09	10

#	Answer	%	Count
1	Definitely not	0.00%	0
2	Probably not	0.00%	0
3	Might or might not	0.00%	0
4	Probably yes	10.00%	1
5	Definitely yes	90.00%	9
	Total	100%	10

Q14 - From the introductory video, did you understand what you were asked to do for the study?

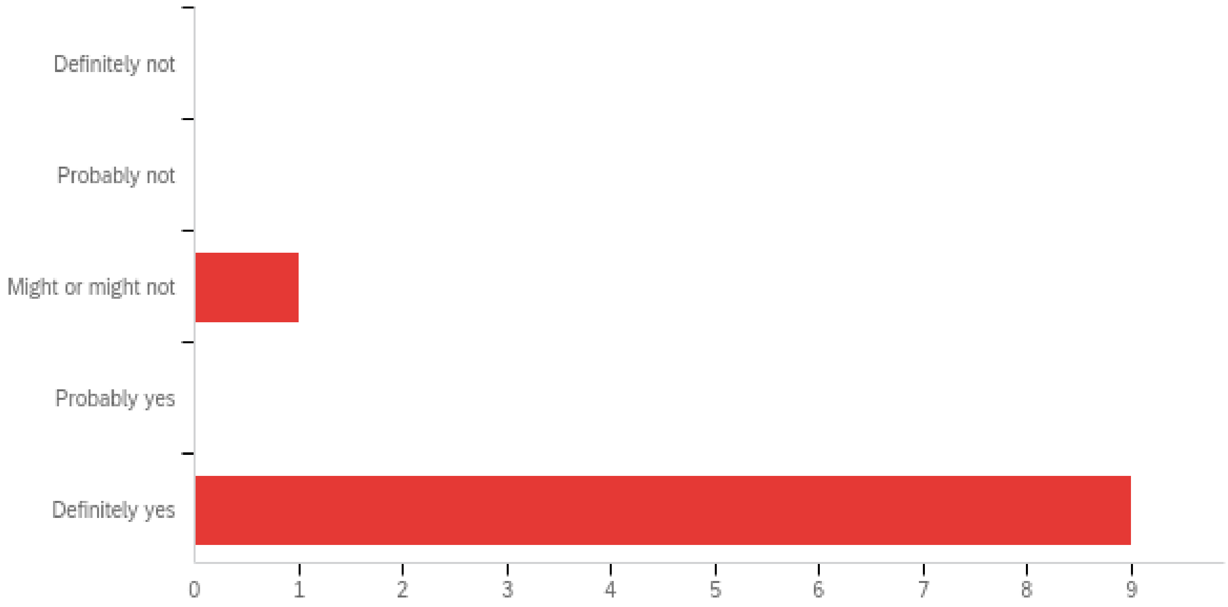

#	Field	Minimum	Maximum	Mean	Std Deviation	Variance	Count
1	From the introductory video, did you understand what you were asked to do for the study?	3.00	5.00	4.80	0.60	0.36	10

#	Answer	%	Count
1	Definitely not	0.00%	0
2	Probably not	0.00%	0
3	Might or might not	10.00%	1
4	Probably yes	0.00%	0
5	Definitely yes	90.00%	9
	Total	100%	10

Q15 - From the introductory video, did you understand how the GIFT system works?

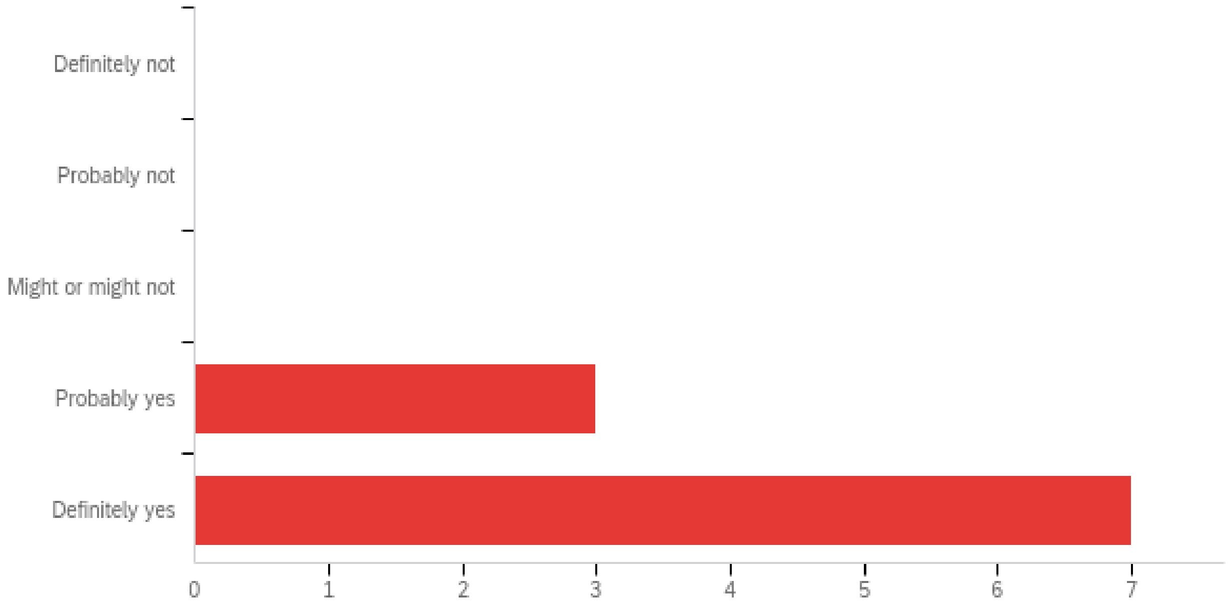

#	Field	Minimum	Maximum	Mean	Std Deviation	Variance	Count
1	From the introductory video, did you understand how the GIFT system works?	4.00	5.00	4.70	0.46	0.21	10

#	Answer	%	Count
1	Definitely not	0.00%	0
2	Probably not	0.00%	0
3	Might or might not	0.00%	0
4	Probably yes	30.00%	3
5	Definitely yes	70.00%	7
	Total	100%	10

Q16 - Did you find the GIFT system easy to use overall?

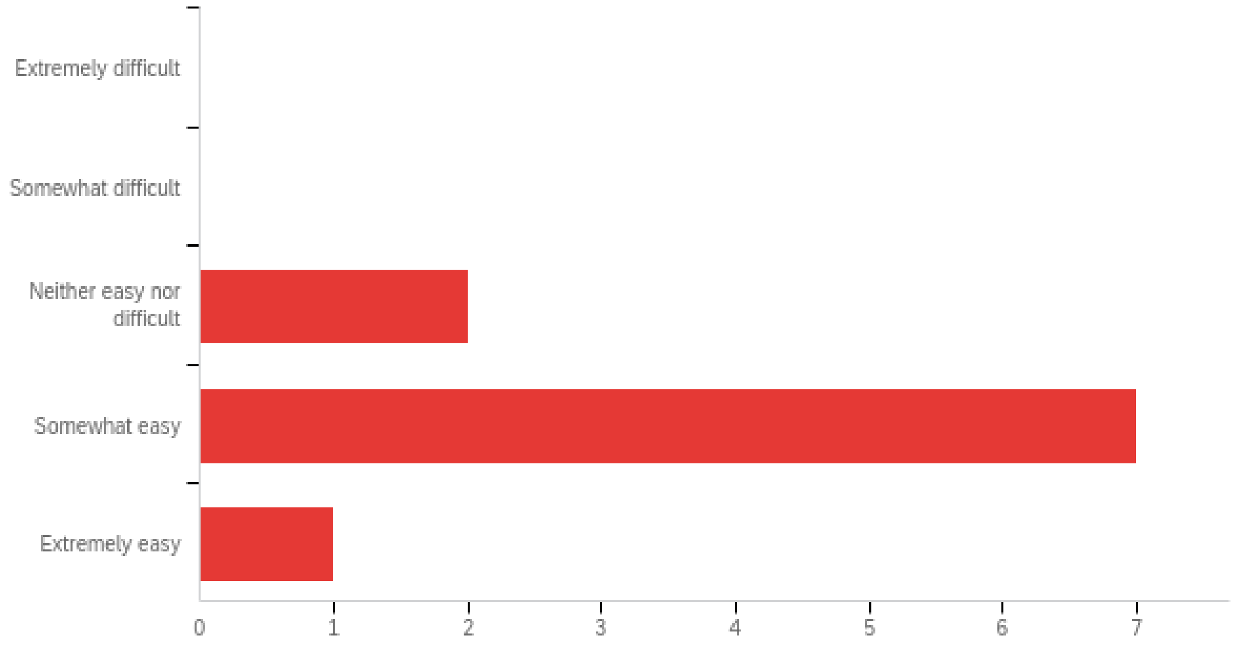

#	Field	Minimum	Maximum	Mean	Std Deviation	Variance	Count
1	Did you find the GIFT system easy to use overall?	3.00	5.00	3.90	0.54	0.29	10

#	Answer	%	Count
1	Extremely difficult	0.00%	0
2	Somewhat difficult	0.00%	0
3	Neither easy nor difficult	20.00%	2
4	Somewhat easy	70.00%	7
5	Extremely easy	10.00%	1
	Total	100%	10

Q17 - If you have any, please tell us your thoughts on the overall system

If you have any, please tell us your thoughts on the overall system
great idea - a little different from normal skin tissue which changed approach. very accurate which made the initial introduction of the needle challenging
Good system, need practice to develop technique, relies on good bronchoscope position
Generally good, gets easier with repeated attempts
There was some glitching in

Q18 - Was the user interface intuitive and easy to understand?

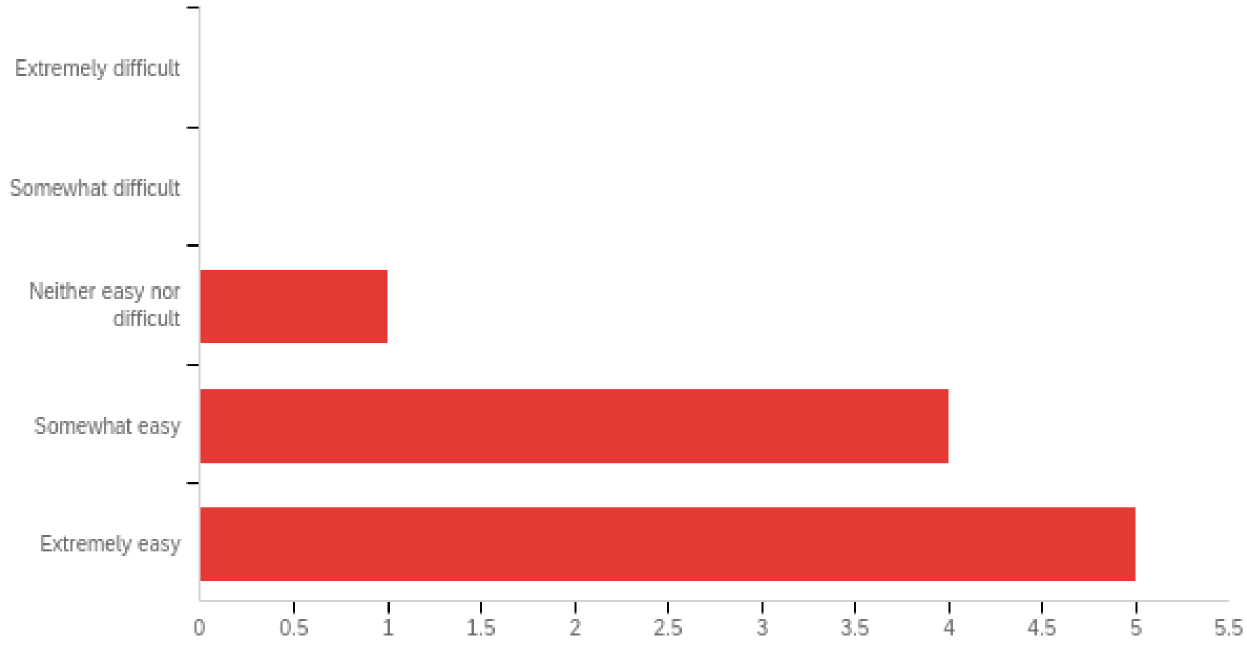

#	Field	Minimum	Maximum	Mean	Std Deviation	Variance	Count
1	Was the user interface intuitive and easy to understand?	3.00	5.00	4.40	0.66	0.44	10

#	Answer	%	Count
1	Extremely difficult	0.00%	0
2	Somewhat difficult	0.00%	0
3	Neither easy nor difficult	10.00%	1
4	Somewhat easy	40.00%	4
5	Extremely easy	50.00%	5
	Total	100%	10

Q19 - If you have any, please tell us your thoughts on how we can improve the user interface

If you have any, please tell us your thoughts on how we can improve the user interface
Indication of which direction/ angular ion would be useful too
Position of the screen so operator movements are matched to movements on screen

Q20 - Did the system physically interfere with any of the existing equipment during the procedure? OR did it physically interfere with yourself?

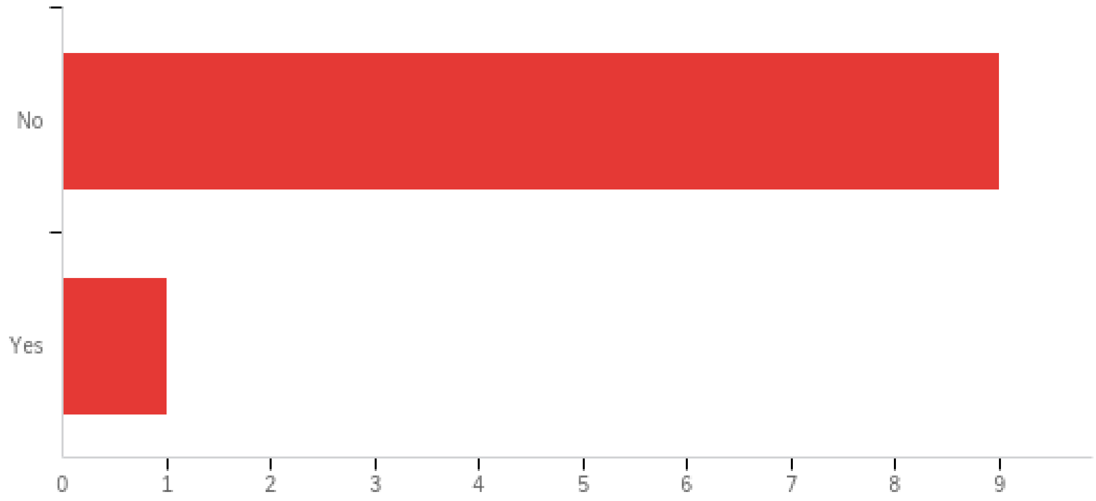

#	Answer	%	Count
1	No	90.00%	9
2	Yes	10.00%	1
	Total	100%	10

Q21 - If yes, how did it interfere?

If yes, how did it interfere?
not so keen on the vibrating band - distracting
I find items on my wrists distracting and am concerned about having vibration/haptic on either of my hands as they are both important in precision of the procedure. I worry that my ‘haptic hand’ may move and reduce this precision.

Q22 - Please rank the feedback type from most intuitive (1) to least intuitive (3)

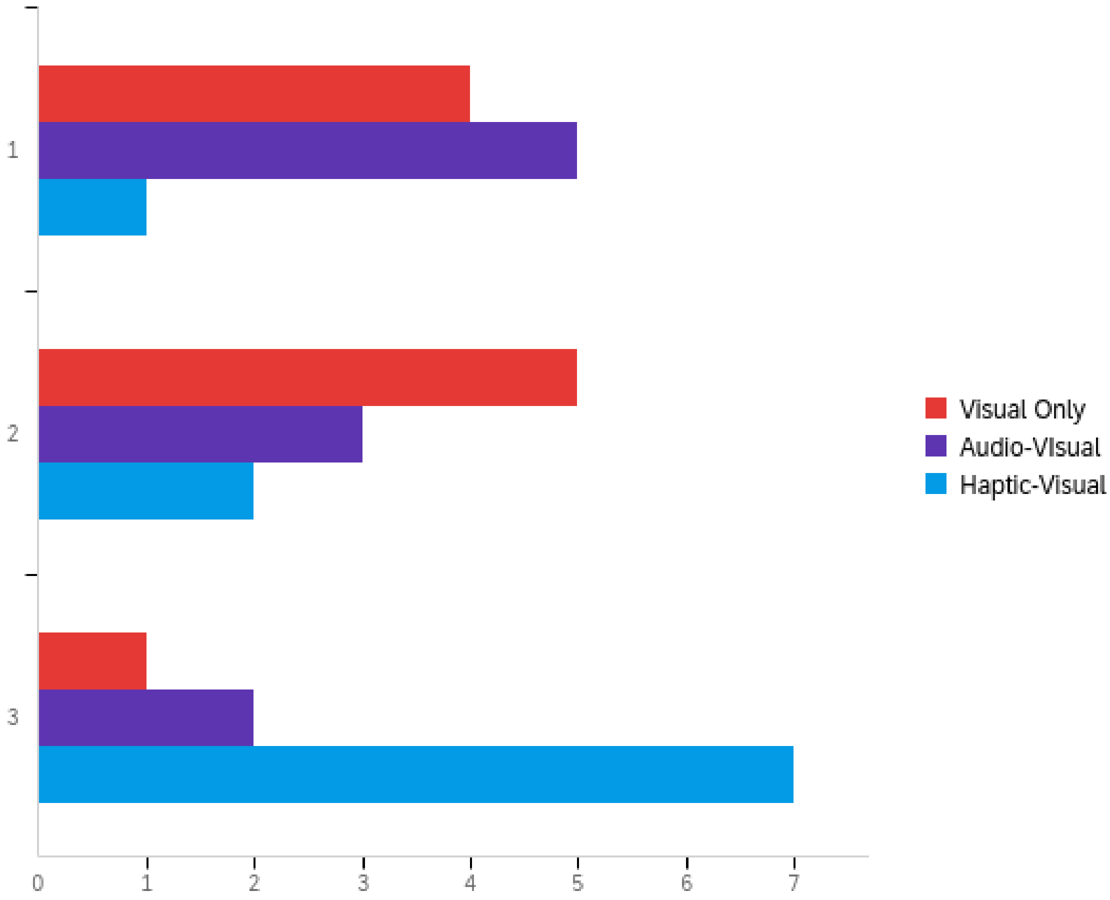

#	Field	Minimum	Maximum	Mean	Std Deviation	Variance	Count
1	Visual Only	1.00	3.00	1.70	0.64	0.41	10
2	Audio-VIsual	1.00	3.00	1.70	0.78	0.61	10
3	Haptic-Visual	1.00	3.00	2.60	0.66	0.44	10

#	Question	1		2		3		Total
1	Visual Only	40.00%	4	50.00%	5	10.00%	1	10
2	Audio-VIsual	50.00%	5	30.00%	3	20.00%	2	10
3	Haptic-Visual	10.00%	1	20.00%	2	70.00%	7	10

Q23 - Please rank the feedback type from most informative (1) to least informative (3)

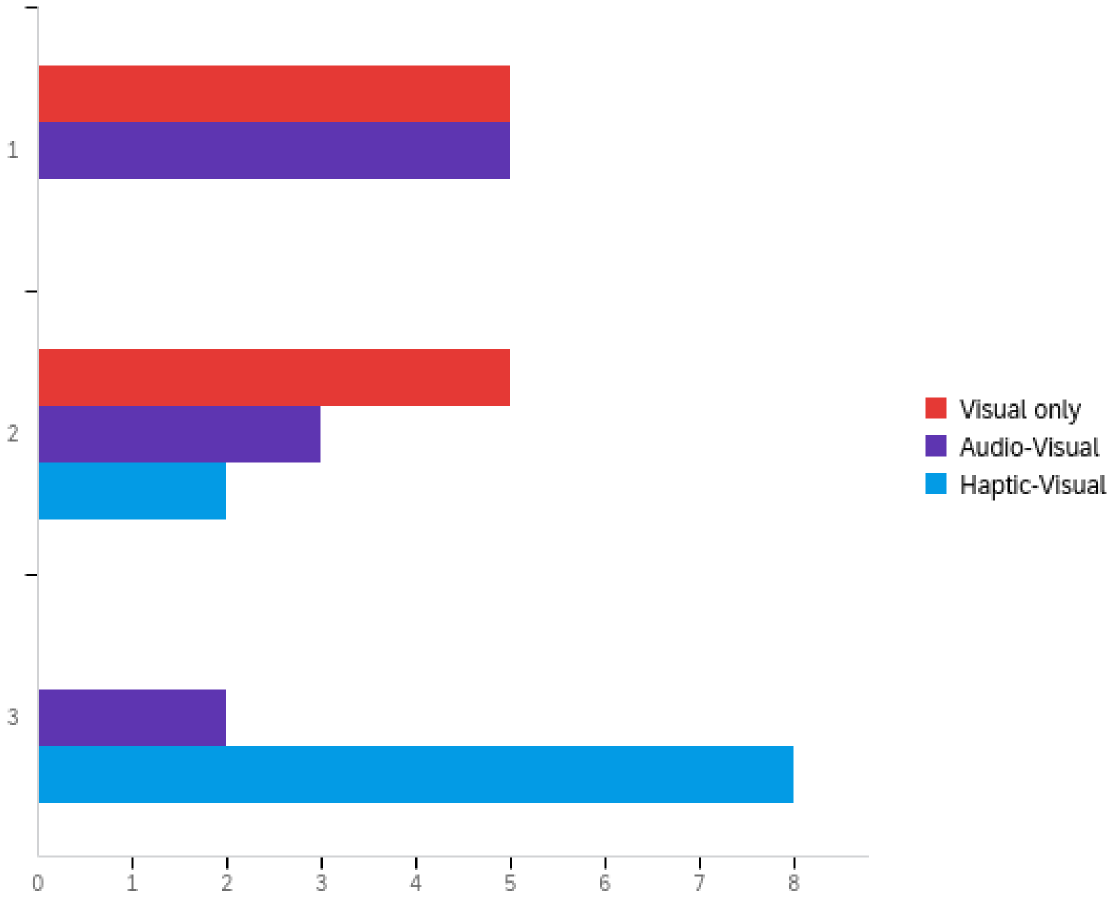

#	Field	Minimum	Maximum	Mean	Std Deviation	Variance	Count
1	Visual only	1.00	2.00	1.50	0.50	0.25	10
2	Audio-Visual	1.00	3.00	1.70	0.78	0.61	10
3	Haptic-Visual	2.00	3.00	2.80	0.40	0.16	10

#	Question	1		2		3		Total
1	Visual only	50.00%	5	50.00%	5	0.00%	0	10
2	Audio-Visual	50.00%	5	30.00%	3	20.00%	2	10
3	Haptic-Visual	0.00%	0	20.00%	2	80.00%	8	10

Q24 - Please rank the feedback type from most enjoyable (1) to least enjoyable (3) to use

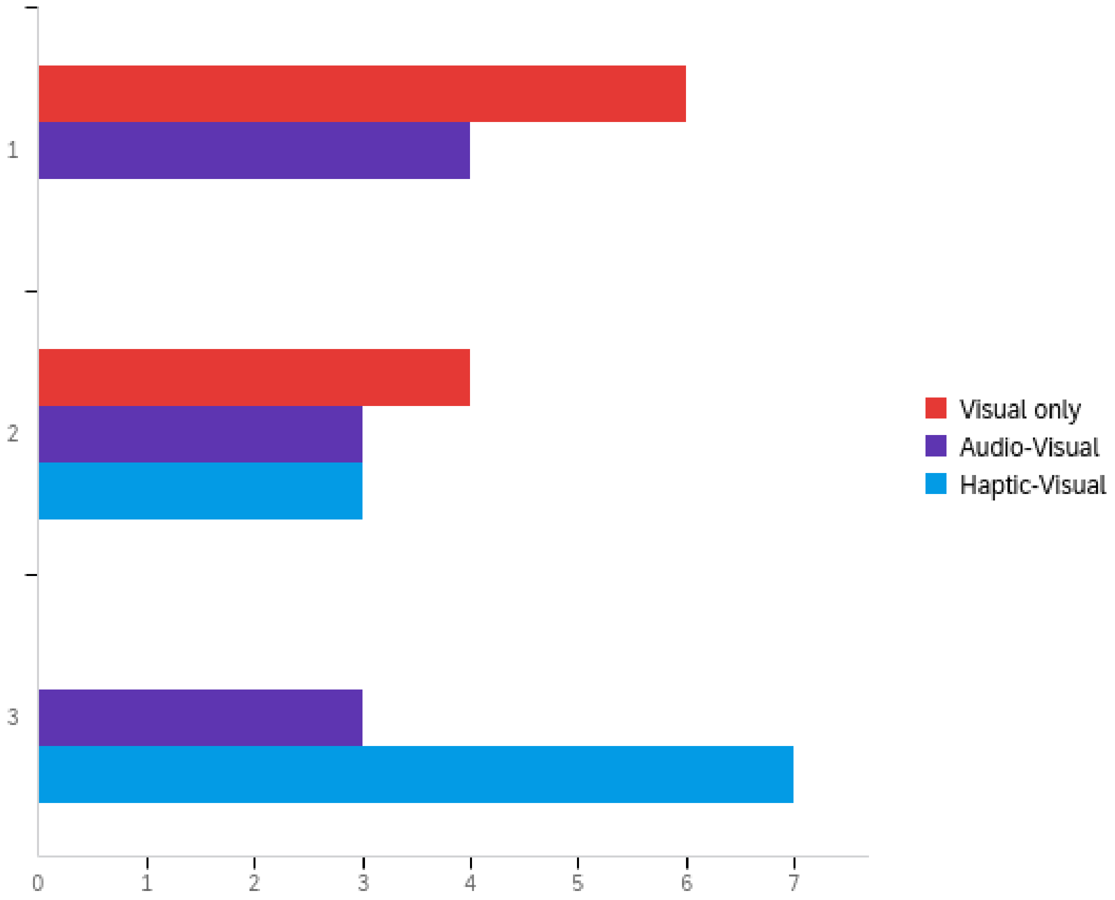

#	Field	Minimum	Maximum	Mean	Std Deviation	Variance	Count
1	Visual only	1.00	2.00	1.40	0.49	0.24	10
2	Audio-Visual	1.00	3.00	1.90	0.83	0.69	10
3	Haptic-Visual	2.00	3.00	2.70	0.46	0.21	10

#	Question	1		2		3		Total
1	Visual only	60.00%	6	40.00%	4	0.00%	0	10
2	Audio-Visual	40.00%	4	30.00%	3	30.00%	3	10
3	Haptic-Visual	0.00%	0	30.00%	3	70.00%	7	10

Q25 - Please rank the feedback type from most stressful (1) to least stressful (3) to use

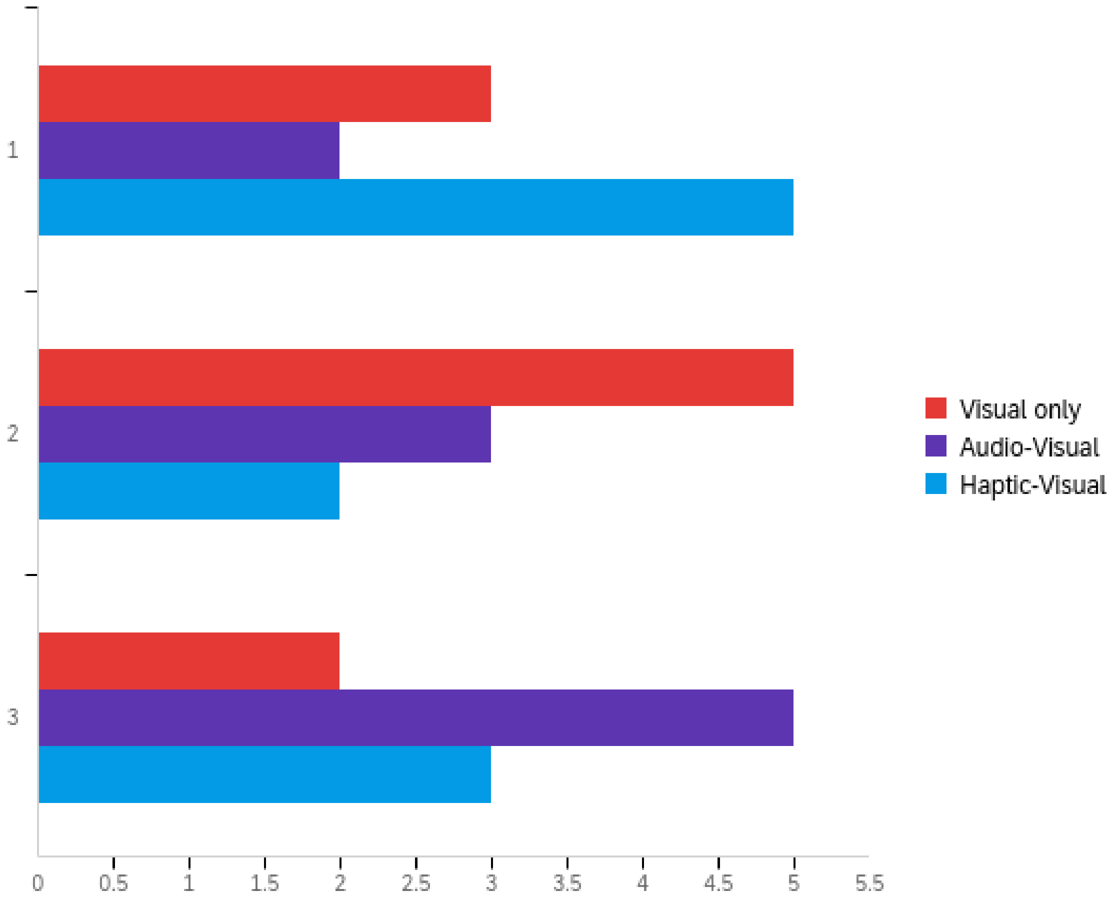

#	Field	Minimum	Maximum	Mean	Std Deviation	Variance	Count
1	Visual only	1.00	3.00	1.90	0.70	0.49	10
2	Audio-Visual	1.00	3.00	2.30	0.78	0.61	10
3	Haptic-Visual	1.00	3.00	1.80	0.87	0.76	10

#	Question	1		2		3		Total
1	Visual only	30.00%	3	50.00%	5	20.00%	2	10
2	Audio-Visual	20.00%	2	30.00%	3	50.00%	5	10
3	Haptic-Visual	50.00%	5	20.00%	2	30.00%	3	10

Q26 - Please rank the procedures from easiest to perform (1) and most difficult to perform (4)

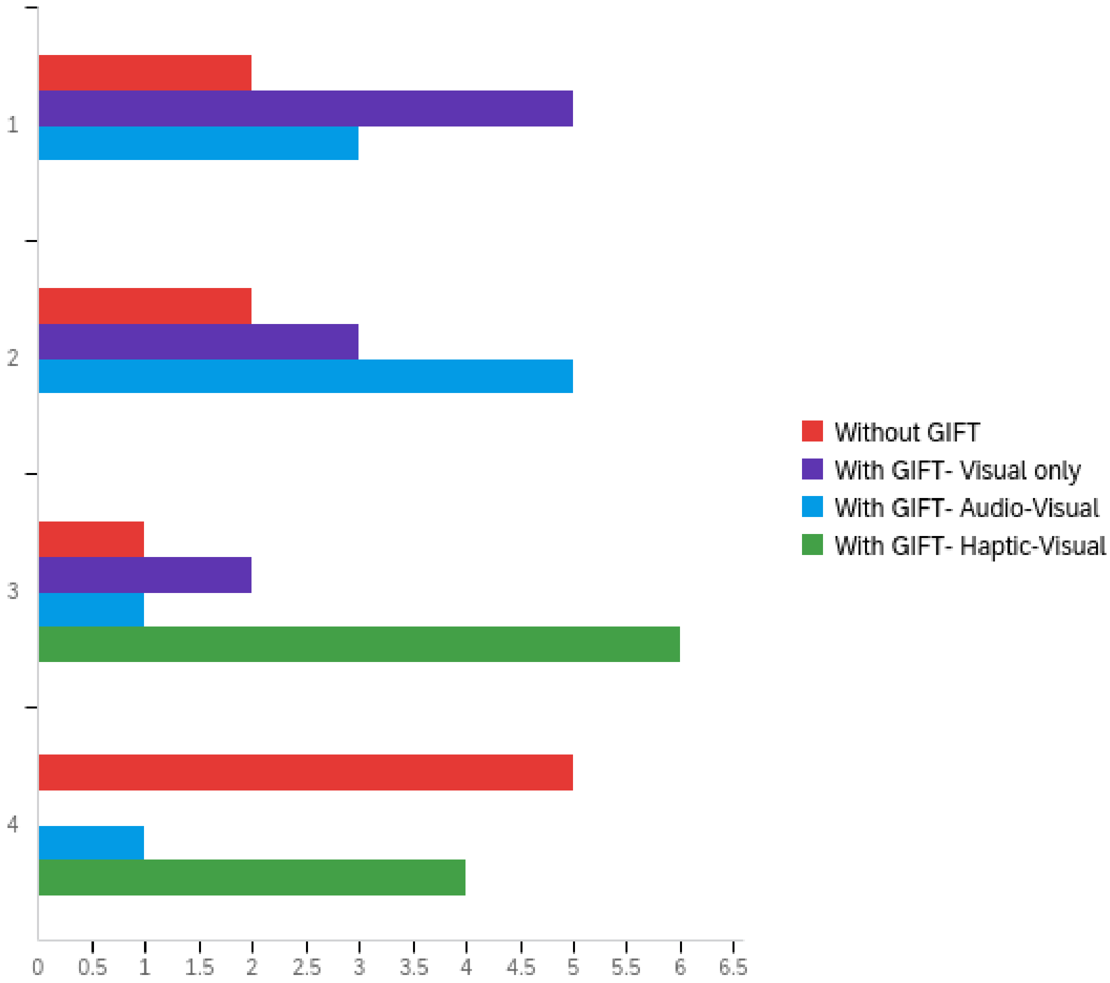

#	Field	Minimum	Maximum	Mean	Std Deviation	Variance	Count
1	Without GIFT	1.00	4.00	2.90	1.22	1.49	10
2	With GIFT- Visual only	1.00	3.00	1.70	0.78	0.61	10
3	With GIFT- Audio-Visual	1.00	4.00	2.00	0.89	0.80	10
4	With GIFT- Haptic-Visual	3.00	4.00	3.40	0.49	0.24	10

#	Question	1		2		3		4		Total
1	Without GIFT	20.00%	2	20.00%	2	10.00%	1	50.00%	5	10
2	With GIFT- Visual only	50.00%	5	30.00%	3	20.00%	2	0.00%	0	10
3	With GIFT- Audio-Visual	30.00%	3	50.00%	5	10.00%	1	10.00%	1	10
4	With GIFT- Haptic-Visual	0.00%	0	0.00%	0	60.00%	6	40.00%	4	10

Q27 - If any, could you tell us what are the benefits and drawbacks for the visual only feedback?

If any, could you tell us what are the benefits and drawbacks for the visual only feedback?
patient safety
allows focus - no additional alarm
Audio feedback also helpful
Simpler, less sources of information
Direct visualisation
Fewer distractions, focus on only one feedback modality
Very easy to understand. Not distracting
There was some glitching of the visual feedback but it was really clear and easy to interpret on the screen

Q28 - If any, could you tell us what are the benefits and drawbacks for the audio-visual feedback?

If any, could you tell us what are the benefits and drawbacks for the audio-visual feedback?
allows better selection of patients
alarms may distract to the audio guide
No drawbacks
There are already lots of alarms on the unit
Good feedback
A reminder if you stray off target while not focused on the visual
Additional cue but only a marginal gain. Extra noise in an already noisy environment probably not useful
Audio useful as I am used to this with patient monitoring

Q29 - If any, could you tell us what are the benefits and drawbacks for the haptic-visual feedback?

If any, could you tell us what are the benefits and drawbacks for the haptic-visual feedback?
vibrations are distracting
would find it distracting
Haptic feedback didn't really guide my needle insertion
New form of feedback, however I liked it
Reinforcing when in the correct position
As for auditory but less effective
Slightly confusing and distracting
I think this could risk dislodging my non-dominant hand and I failed to interpret it as easily. It is not often used in other parts of our practice.

Q30 - Is there anything else you would like to use to know or can be improved with the overall system?

Is there anything else you would like to use to know or can be improved with the overall system?
better targeting with the two sensors
Changing visual interface to guide angular ion of needle insertion
I like the idea. It takes time to get used to the angle of approach. Not sure it’s easy to consistently stay in the circle. But I like the reassurance it gives
Bronchoscope/sensor position
Nothing to help you avoid tracheal rings, some occasional jumpy movement of visual despite no movement of needle
No
Small amount of glitching in the software
